# Concurrent Temperature and Light Intensity Fluctuations Promote Stomatal Opening and Non‐Steady State Photosynthesis

**DOI:** 10.1111/pce.70509

**Published:** 2026-04-02

**Authors:** Samikshya Shrestha, Elias Kaiser, Sarah R. Berman, Maarten L. J. Wassenaar, Tom van den Berg, Evi Mavrou, Leo F. M. Marcelis, Silvere Vialet‐Chabrand

**Affiliations:** ^1^ Horticulture and Product Physiology, Plant Science Group Wageningen University and Research Wageningen the Netherlands; ^2^ Research, Institute of Agriculture and Life Sciences Seoul National University Seoul Republic of Korea

**Keywords:** acclimation, fluctuating light, leaf temperature, net CO2 assimilation, stomatal conductance

## Abstract

In nature, fluctuations in light intensity (FL) are tightly coupled to rapid changes in leaf temperature (*T*_*leaf*_), yet the short‐term physiological effects of these concurrent drivers remain largely unresolved. Here, we combined rapid infrared induced *T*_*leaf*_ fluctuations with controlled step changes in light intensity for leaf gas exchange measurements to disentangle how each factor, and their interaction, affects stomatal conductance (*g*_*s*_) and net CO_2_ assimilation (*A*) in cucumber plants grown under constant light or FL. Modest but rapid increases in *T*_*leaf*_ (~3°C) alone triggered a pronounced wrong‑way–like stomatal response, leaf movements due to change in epidermal cell turgor, and transient (but long‐lasting) decrease in *A*. This transient decrease in *A* disappeared under low O₂, indicating a photorespiratory origin. When *T*_*leaf*_ and light increased simultaneously, both *g*_*s*_ and *A* responded faster and more strongly than to light alone, with cumulative enhancement across successive cycles. Plants acclimated to FL displayed larger transient increases in *A* and maintained higher integrated carbon gain under combined fluctuations. Our findings demonstrate that realistic, rapid *T*_*leaf*_ dynamics exert strong biomechanical and biochemical influences on dynamic gas exchange that are distinct from steady‐state responses and should be explicitly considered in experimental design and dynamic photosynthesis models.

## Summary statement

Rapid and simultaneous increases in leaf temperature and light intensity caused faster and stronger stomatal opening and photosynthetic induction.

## 1 Introduction

In nature, leaves frequently experience fluctuations in light intensity (FL) due to constant changes in solar position, clouds, and wind (Pearcy et al. 1997; Way and Pearcy 2012). Naturally, these fluctuations result in concurrent and rapid changes in leaf temperature (*T*_*leaf*_) of up to 10°C (Singsaas and Sharkey 1998; Leakey et al. 2003) and changes in leaf‐to‐air vapor pressure difference (VPD). Net CO_2_ assimilation (*A*) and stomatal conductance (*g*_*s*_) respond continuously to these fluctuations, causing rapid changes in this ratio, also known as intrinsic water use efficiency (iWUE; Lawson and Blatt 2014; McAusland et al. 2016). However, previous literature focused only on the effects of FL (Way and Pearcy 2012; Kaiser et al. 2015), although processes such as the activation of Calvin cycle enzymes and stomatal movement also depend on temperature, but their role is overlooked (Way and Pearcy 2012; Kaiser et al. 2015). A rapid increase in *T*_*leaf*_ simultaneously with light intensity might create imbalances in the metabolic flux, thereby impacting *A*, *g*_*s*_, and photorespiration. While few studies have explored the effects of stable *T*_*leaf*_ on photosynthetic induction (Küppers and Schneider 1993; Leakey et al. 2003; Kaiser et al. 2015, 2017; Wachendorf and Küppers 2017; Zheng et al. 2022) and stomatal conductance (Raschke 1970; Rogers et al. 1979; Kostaki et al. 2020; Korte et al. 2023), the effects of single *T*_*leaf*_ or combined *T*_*leaf*_ and light intensity step changes have, to our knowledge, only been investigated twice (Kang et al. 2020; Pankasem et al. 2024). However, these studies only imposed gradual changes in *T*_*leaf*_ due to technical limitations in the gas exchange system, and the effects of rapid concurrent changes in light intensity and *T*_*leaf*_ on *A* and *g*_*s*_ have not been explored. Since FL frequently occurs in nature (Mol et al. 2023), so do *T*_*leaf*_ fluctuations (Leuzinger and Körner 2007; Fauset et al. 2018), potentially impacting plant gas exchange in the field.

Changes in *T*_*leaf*_ (and thus VPD) can change membrane fluidity and permeability (Ilan et al. 1995). These changes may further cause mechanical and/or osmotic changes in the stomatal guard cells and the cells that surround them, depending on the need for evaporative cooling and water status. While stomata open in response to increases in light intensity and *T*_*leaf*_ (Merilo et al. 2014; Pankasem et al. 2024), stomatal responses to VPD are less straightforward: after a step increase in VPD (e.g. > 1 kPa; Merilo et al. 2014; Zait et al. 2024) or after leaf excision (Buckley 2005; Buckley et al. 2011) a transient opening is often observed (2‐20 min) that is known as the “wrong‐way” response (WWR) or “Iwanoff effect,” which is typically followed by a reduction in stomatal aperture. The WWR is caused by a sudden drop in the turgor of the epidermal cells surrounding the guard cells, thus forcing the stomata to open transiently (Buckley 2005). Stomatal responses to combinations of several factors can be complex: Merilo et al. (2014) showed that stomatal responses to two simultaneously applied but opposing factors were species‐specific and could not easily be predicted from single‐factor responses. In another study in which two environmental factors were simultaneously changed, hydraulic factors (leaf water potential and air humidity) were observed to dominate the stomatal response over photosynthesis‐related factors (light and CO_2_), and stomatal responses were non‐additive (Aasamaa and Sober 2011). However, when two photosynthesis‐related factors that affected *g*_*s*_ in the same direction were changed, the response was additive (Aasamaa and Sober 2011). Since both light and temperature have been shown to positively affect *g*_*s*_, it can be hypothesized that their effects are also additive, but this has not been tested.

Both stomatal and Calvin cycle enzyme kinetics limit *A* induction under FL (Way and Pearcy 2012; Kaiser et al. 2015), and these processes are affected by temperature and VPD (Kaiser et al. 2017). The activity of photosynthetic biochemistry increases with temperature up to an optimum; for example, high temperatures inhibit photosynthesis by reducing the activation of Rubisco by Rubisco activase (Salvucci et al. 2001). While no effect of steady‐state *T*_*leaf*_ on photosynthetic induction was observed by Leakey et al. (2003) in *Shorea leprosula*, other studies have shown acceleration of photosynthetic induction under increased *T*_*leaf*_ (Kaiser et al. 2017; Wachendorf and Küppers 2017; Zheng et al. 2022). Briefly, the effects observed in these studies were: faster *A* induction (*Solanum lycopersicum*, Kaiser et al. 2017; several tree species, Wachendorf and Küppers 2017; *Glycine max*, Zheng et al. 2022), and accelerated stomatal opening (Wachendorf and Küppers 2017; Zheng et al. 2022). Concurrent increases in *T*_*leaf*_ and light intensity caused a faster increase in *A* in tropical tree species compared to constant *T*_*leaf*_ (Kang et al. 2020), but this is the only study showing this effect. Overall, increasing temperature during a step change in light intensity was observed to increase the turnover rates of photosynthetic biochemistry and to contribute to stomatal opening. Changes in photorespiratory metabolite pools and rate of CO_2_ diffusion during photosynthetic induction also affect photorespiration, which, in turn, was suggested to interact with photosynthetic induction (Fu and Walker 2023).

When studying temperature responses using a gas exchange system, most studies rely on controlling air temperature to control *T*_*leaf*_. However, this often leads to artefactual data after a step change due to slow equilibration times of the new conditions in the measuring chamber (Pankasem et al. 2024). Authors often remove *g*_*s*_ data immediately after step changes in temperature or VPD, thus ignoring this part of the response (Mott and Peak 2010; Wang et al. 2017; Pankasem et al. 2024; Zait et al. 2024). However, as suggested by Mott and Peak (2013), *T*_*leaf*_ can be altered rapidly using infrared radiation. This technique can provide rapid temperature changes without artefacts, and may allow us to observe stomatal responses to *T*_*leaf*_.

Leaves grown under FL acclimate to the environment through changes in morphology, anatomy, and physiology (Vialet‐Chabrand et al. 2017; Durand et al. 2024; Shrestha et al. 2025), some of which may interact with *T*_*leaf*_ fluctuations. Acclimation to FL was sometimes shown to accelerate the kinetics of *A* and *g*_*s*_ (Kaiser et al. 2018; Matthews et al. 2018; Qiao et al. 2021) and reduce photorespiration (von Bismarck et al. 2023) compared to acclimation to non‐fluctuating light. Since plants grown under FL also experience fluctuations in *T*_*leaf*_, part of their acclimation response may be driven by temperature variations. Therefore, FL‐grown plants could be able to better cope with rapid *T*_*leaf*_ fluctuations. It is unknown how acclimation to FL impacts on leaf physiology under short‐term temperature fluctuations.

To understand the effects of rapid temperature changes on leaf gas exchange, we assessed the transient responses of leaves to *T*_*leaf*_ and/or light intensity fluctuations in cucumber plants that had been grown under constant light intensity (hereafter: square wave, SQ) or under light that alternated between low and high intensities (hereafter: alternating, AL). We asked 1) whether acclimation to AL affects the response of gas exchange to light and *T*_*leaf*_ fluctuations, 2) how rapid *T*_*leaf*_ step changes affect *g*_*s*_ and *A*, and 3) whether rapid *T*_*leaf*_ increases promote faster and larger gas exchange responses to light intensity increases. We hypothesized that 1) AL‐grown plants acclimate to concurrent fluctuations in light intensity and *T*_*leaf*_, enhancing the magnitude and rapidity of g_s_ and *A* responses, 2) step changes in *T*_*leaf*_ only (compared to constant *T*_*leaf*_) promote stomatal opening and increase *A*, and 3) when both *T*_*leaf*_ and light intensity are concurrently increased, *g*_*s*_ and *A* respond more quickly and with a larger magnitude than when *T*_*leaf*_ is kept constant during FL.

## 2 Materials and Methods

### 2.1 Plant Material and Growth Conditions

The experiment was conducted in a custom‐made climate container comprised of nine compartments of 1.3 × 1.0 m each. Cucumber seeds (*Cucumis sativus* cv. Hi‐Power, Nunhems BASF, the Netherlands) were sown in rockwool plugs (2 cm diameter; Grodan, Roermond, the Netherlands). Seven days after sowing, seedlings were transplanted to rockwool blocks (10 × 10 x 6.5 cm; Grodan, Roermond, the Netherlands) and grown for 4 weeks under two light treatments. Eight plants were grown per compartment at a density of 6.2 plants m^−2^. Side shoots were removed when they were < 2 cm long. Plants were irrigated once every 2 days during the first 2 weeks of the experiment and daily thereafter with nutrient solution (pH 5.8, EC 2.4 dS m^−1^) in an automatic ebb and flood system. The nutrient solution was composed of NH_4_^+^ 0.5 mM L^−1^, K^+^ 8.63 mM L^−1^, Ca^2+^ 4.91 mM L^−1^, Mg^2+^ 2.18 mM L^−1^, NO_3_^−^ 14.6 mM L^−1^, SO_4_^2−^ 4.02 mM L^−1^, H_2_PO_4_^−^ 1.37 mM L^−1^, Mn 10 µM L^−1^, Zn 5 µM L^−1^, B 30 µM L^−1^, Cu 0.75 µM L^−1^, Mo 0.5 µM L^−1^, and Fe 25 µM L^−1^.

Growth conditions were 22.3 ± 0.6/19.7 ± 0.17°C day/night temperature, 433.6 ± 69.3 ppm CO_2_, 73.1 ± 1.6% relative humidity (RH), air flow 0.34 ± 0.04 m s^−1^, and 16 h photoperiod (07.00 until 23.00). Light was provided by dimmable LED lamps (HORTILED Multi 4DIM, 120 cm, Red/Blue/White/FR; HORTILUX SCHRÉDER, the Hague, the Netherlands) with a spectral composition of 25% blue, 9% green, 66% red, and 9% far‐red of the total PPFD. After 3 weeks of treatment, leaf #4 (as counted from the bottom) was used for gas exchange measurements. Plants were supported with wooden sticks 3‐4 days before the start of gas exchange measurements (Supporting Information Figure S1), such that the majority of leaf #4 was stably exposed to the light treatment prior to gas exchange measurements.

### 2.2 Growth Light Treatments

Plants were grown under two light regimes during the photoperiod: (1) a square wave light regime (SQ) with a constant light intensity of 275 µmol m^−2^ s^−1^, and (2) alternating light intensities (AL), which changed between 50 and 500 µmol m^−2^ s^−1^ every 15 min (Supporting Information Figure S2). Plants in both treatments received the same average light intensity, resulting in a daily light integral of 15.84 mol m^−2^ d^−1^. Plants within each compartment were randomized every 3 days to correct for spatial variabilities.

### 2.3 Measurements

#### 2.3.1 Leaf Temperature Under AL Growth Treatment

Under the growth conditions of the AL treatment, leaf temperature kinetics showed a larger response to changes in light intensity compared to those measured in a gas exchange cuvette, due to differences in energy balance (e.g. boundary layer conductance, cuvette wall temperature emission; (Still et al. 2019)). To reproduce *T*_*leaf*_ kinetics in gas exchange measurements close to those experienced by plants under growth conditions (see below), we monitored *T*_*leaf*_ under AL growth treatment. A type K thermocouple (RS PRO Type K Exposed Junction Thermocouple 2 m length, 7/0.2 mm diameter; RS Pro; UK) was placed carefully at the bottom surface of leaf #4 to record leaf temperature kinetics for 8 h. In addition, a black tape with similar longwave emissivity (0.97) and short‐wave absorptance (0.96 in the range 400−700 nm) as a leaf was equipped with a type K thermocouple and placed close to leaf #4 to characterize the energy required to increase *T*_*leaf*_ without the effect of variable leaf evaporative cooling (Vialet‐Chabrand and Lawson 2019). The black tape was folded around the thermocouple with the hard core wire providing support, and the thin tape ensuring a fast response to temperature changes due to changes in illumination.

#### 2.3.2 Photosynthetic Gas Exchange: Light Intensity and Temperature Fluctuation Protocols

Leaf gas exchange measurements were started 21 days after the start of growth light treatments and were finished within 5‐6 days. Measurements were done using the 9 cm^2^ clear‐top chamber (6800‐12 A) of the LI‐6800 photosynthesis system (LI‐COR Biosciences, Lincoln, NE, USA). Preliminary tests showed that *T*_*leaf*_ inside the cuvette increased by only ~0.6°C after a step change in light intensity (45–445 μmol m^−2^ s^−1^) by an external LED lamp (ELIXIA, Heliospectra, Gothenburg, Sweden), when temperature of the heat exchanger (T_xchg_) was controlled. However, under the AL treatment, *T*_*leaf*_ changed by ~2°C under similar light intensity changes (Supporting Information Figure S3, S4), suggesting that *T*_*leaf*_ changes were strongly dampened in the LI‐6800 clear‐top cuvette. We used an infrared (IR) lamp (Burda Term 2000 IP67 1.65 kW Low Glare; Burda, Enschede, the Netherlands) to rapidly heat the leaf within seconds; this cannot be achieved using the Peltier element of the LI‐6800. To emulate the *T*_*leaf*_ kinetics observed under AL inside the gas exchange cuvette, the same black tape was placed inside the cuvette, and T_xchg_ was controlled at 21°C. The IR lamp was only switched on during the high temperature phase, and setpoints to produce similar *T*_*leaf*_ changes as observed under AL were achieved by manually adjusting its intensity. IR lamp setpoints were also tested with leaves placed in the cuvette to ensure that temperature kinetics matched those observed under growth conditions.

The intensity of the IR lamp was controlled via a power controller (M240, Kemo Electronic GmbH, Geestland, Germany) which was interfaced using the pulse width modulation (PWM) output of an Arduino Uno R4 (Arduino, Monza, Italy). Fluctuations in light intensity were provided by an LED lamp (ELIXIA, Heliospectra, Gothenburg, Sweden), with a spectral composition of 22% blue, 12% green, 66% red, and 9% far‐red. The duty cycle of the PWM output as well as the setpoints of the LED lamp were controlled via software according to the measurement protocol to expose leaves to controlled fluctuations in temperature and light. Both IR and LED lamps were positioned close to each other atop a metal frame (Supporting Information Figure S1). The LI‐6800 cuvette was placed directly below the infrared lamp, so that the leaf surface was exposed to both light and temperature fluctuations. All other parts of the LI‐6800 were covered with heat‐reflective plastic to avoid heating of the machine (Supporting Information Figure S5b). The complete setup was placed in the climate container and covered on two sides to avoid light contamination from the growth compartments. Two fans (San Ace B97, 9BMB12P2F01, Sanyo Denki Co., LTD, Singapore) were installed at the opposite side of the infrared heater to provide air circulation and reduce temperature heterogeneity.

Environmental setpoints for the LI‐6800—other than light intensity and *T*_*leaf*_ (see below)—during photosynthesis measurements were 450 µmol mol^−1^ [CO_2_], 65% RH, and 500 µmol s^−1^ flow rate. Infra‐red gas analyzers (IRGAs) were matched 0, 35, and 65 min after the start of the protocol. The same LI‐6800 instrument was used for all light intensity and *T*_*leaf*_ protocols. Measurements were conducted between 08:00 and 15:00, to reduce diurnal effects on gas exchange measurements. No plants were repeatedly measured between gas exchange protocols. Plants were well watered throughout measurements.

**Figure 1:**
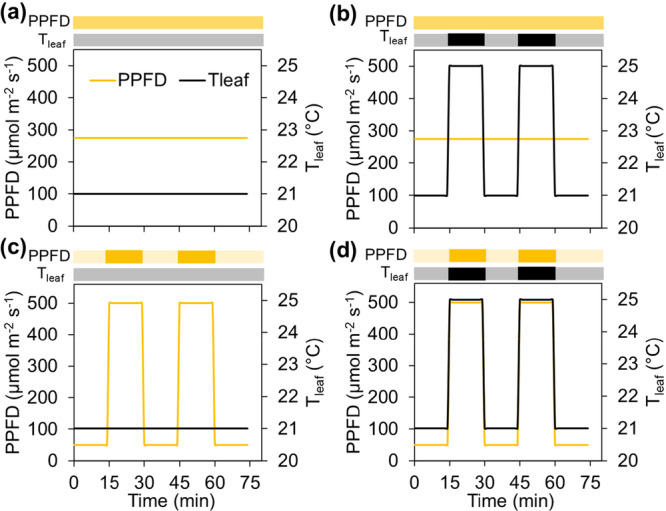
Schemes of constant and fluctuating light (PPFD) and leaf temperature (*T*_*leaf*_) protocols: (a) constant light + constant temperature (CL + CT), (b) constant light + fluctuating temperature (CL + FT), (c) fluctuating light + constant light (FL + CT), and (d) fluctuating light + fluctuating temperature (FL + FT). Yellow and gray bars above panels represent PPFD and *T*_*leaf*_; bars with uniform color represent constant PPFD and/or *T*_*leaf*_, bars alternating between light and dark shades indicate fluctuations in PPFD and/or *T*_*leaf*_.

Four protocols were used: (1) constant light and temperature (CL + CT), (2) constant light and fluctuating temperature (CL + FT), (3) fluctuating light and constant temperature (FL + CT), and (4) fluctuating light and temperature (FL + FT; Figure 1; Table S1). Initially, the set points for the LED lamp for the light regimes used in the gas exchange protocols were 286 µmol m^−2^ s^−1^ for CL and fluctuating between 52 and 520 µmol m^−2^ s^−1^ for FL. We systematically recorded light intensity within the cuvette using the GaAsP sensor readings for each measurement as small position changes of the cuvette can impact light intensity readings. As the GaAsP sensor has different spectral sensitivity to a PAR sensor and is not cosine corrected, the readings were corrected by calibrating the GaAsP sensor against a PAR sensor (LI‐190R, LI‐COR Biosciences) that was placed close to it inside the LI‐6800 cuvette. After correction, the light intensities (mean ± SD) achieved for the different measurement protocols were: 321.6 ± 3.4 µmol m^−2^ s^−1^ for CL + CT, 320.5 ± 4.4 µmol m^−2^ s^−1^ for CL + FT, 49.3 ± 1.2 and 532.5 ± 6.5 µmol m^−2^ s^−1^ for FL + CT, and 49.6 ± 1.1 and 546.9 ± 11.5 µmol m^−2^ s^−1^ for FL + FT. The FL pattern used here was similar to AL at the level of leaf #4 during growth; nevertheless, we deliberately used different names to differentiate between long‐term growth treatment and short‐term gas exchange protocol. For CT, *T*_*leaf*_ was 21°C while for FT, *T*_*leaf*_ fluctuated between 21 and ~23.5–24.5°C. In case of fluctuating measurement regimes, measurements always started with low light intensity and/or *T*_*leaf*_. This was followed by two successive cycles of step increases to high light intensity and/or *T*_*leaf*_, followed by step reductions to low light intensity and/or *T*_*leaf*_. Each step lasted 15 min, with the whole protocol lasting 75 min and data was logged every 2 s.

The IR lamp added ~1 µmol m^−2^ s^−1^ of photosynthetically active radiation (PAR; 400−700 nm), and ~3 µmol m^−2^ s^−1^ far‐red light (700–780 nm; measured using the LI‐180 Spectrometer, LI‐COR Biosciences) during high *T*_*leaf*_ phases, which was considered negligible. During measurements, the entire plant was exposed to the measurement protocol (Supporting Information Figure S5b). The leaf inside the cuvette was first acclimated to initial conditions, until *A* and *g*_*s*_ were visibly stable (30–70 min). For the FT protocol, control of *T*_*leaf*_ was used initially, but was then switched to control by T_xchg_ just before starting the protocol, to avoid artefacts from correctional adjustments in temperature or RH (Kang et al. 2020). During the FT protocol, cuvette air temperature changed between 20.93 ± 0.05 and 23.15 ± 0.33°C (mean ± SD), while RH changed by only 0.42 ± 0.20%. Air vapor pressure changed by 0.23 ± 0.03 kPa, while leaf‐to‐air VPD changed by 0.29 ± 0.05 kPa.

#### 2.3.3 Microscopy of Dynamics of Individual Stomata Under CL + FT Measurement Protocol

To further understand *g*_*s*_ responses to CL + FT, microscopy measurements of stomatal dynamics were conducted using a custom microscope described in van den Berg et al. 2025. The microscope was placed in the same setup as for gas exchange measurements. Plants were moved from their growth compartment to the measurement area on the evening before the day of measurement. Leaf #4 was gently clamped in the microscope holder (Supporting Information Figure S6). A second leaf clip from a mini‐PAM (Heinz Walz, Effeltrich, Germany) was clamped nearby the microscope to measure PPFD and *T*_*leaf*_ at the leaf level. The light intensity of the LED lamp was set to 730–740 µmol m^−2^ s^−1^ at leaf level (Supporting Information Table S1). *T*_*leaf*_ increased from 26 ± 0.5°C to 32.5 ± 0.5°C during the first cycle and from 27 ± 0.5°C by 33 ± 1°C during the second cycle (Supporting Information Figure S7). The increased apparent light and temperature changes in the microscopy experiment compared to gas exchange measurements were attributable to the limited optical resolution of the microscope, which was insufficient to resolve subtle aperture changes in the small cucumber stomata at lower light and temperature levels. Airflow, measured with a hot‐sphere anemometer (KANOMAX, Osaka, Japan), was 0.23 m s^−1^ at the top of the leaf, 0.07 m s^−1^ lower than in the growth chamber but well within the range of airflow speeds typically experienced by indoor grown crops (Dupont et al. 2025). Imaging with a 50x objective (Mitutoyo, 50xHR, NA 0.75) started after 60 min of light acclimation. Stacks of 130 images, spaced 0.5 µm apart parallel to the imaging plane, were recorded every minute. After 90 min of light exposure, the CL + FT protocol was started, and imaging continued for 60 min. Linear fits of each 15 min period were made in Origin (OriginLab, Northampton, MA, USA), with slope and intercept as free parameters, to assess the rate of opening or closing of stomatal pores.

#### 2.3.4 Temperature Response Curves

Temperature response curves of *A* and *g*_*s*_ under photorespiratory (21% O_2_) and non‐photorespiratory conditions (2% O_2_) were measured using the LI‐6800 photosynthesis system with the 6800‐01 A fluorometer and an enclosed leaf area of 6 cm^2^. Air with 2% O_2_ was generated by mixing N_2_ and O_2_ from separate gas cylinders (SOL Nederland B.V., Tilburg, The Netherlands) in a ratio of 98:2 using mass flow controllers (Brooks 5850E series, Brooks, Veenendaal, The Netherlands), and gas composition was confirmed using an oxygen analyzer (type 570 A, Servomex, Crowborough, UK). The outlet of the mass flow controllers was attached to the inlet of the LI‐6800 using a T‐piece to prevent pressure‐induced damage to the pump of the LI‐6800, and excess air was provided to ensure that no ambient air was pumped in. When measuring at 2% O_2_, the system constant in the LI‐6800 was set to 2% O_2_ to apply the band‐broadening correction. Environmental conditions were controlled at 300 µmol m^−2^ s^−1^ PPFD, 450 µmol mol^−1^ [CO_2_], VPD of 1 kPa, and flow rate of 400 µmol s^−1^. Light was provided by a mixture of red (90%) and blue (10%) LEDs in the fluorometer. The leaf was first adapted to 18°C air temperature until *A* and g_s_ were visibly stable (60‐70 min). Then, air temperature was increased in steps of 21, 24, 27, and 30°C, using the Peltier element. Data was logged every 2 s for the first 5 min and then after every 10 s for the remaining 25 min, in total 30 min at each temperature. IRGAs were matched at each temperature.

#### 2.3.5 Stomatal Density and Size

Stomatal imprints were taken after gas exchange measurements had been completed on a given day, and at similar spots where gas exchange was measured. Plants were exposed to growth light conditions for ≥ 1 h before imprints were taken. To create imprints, Zhermack elite HD+ silicon (Zhermack SpA, Badia Polesine, Italy) was dotted onto the leaf. Per leaf, four imprints were taken from the bottom and top leaf surface each. Clear nail polish was applied to the impression, peeled off when dry, and viewed under the microscope (Leitz Aristoplan; Leica Microsystems, Wetzlar, Germany). Pictures were taken with the 40x objective (camera: Axiocam 305 color; Carl Zeiss, Oberkochen, Germany), and 11–12 pictures per leaf surface per replicate plant were analyzed using the ObjectJ and CellCounter plugins in ImageJ v1.54p (National Institute of Health, Bethesda, MD, USA). Stomatal density and size were calculated following Savvides et al. (2012).

#### 2.3.6 Biomass and Leaf Area

Plants were harvested destructively 26–27 days after transplanting. Fresh shoot mass and leaf area were measured. Dry weight was measured after drying plant organs in the oven at 70°C for 3 days. 5–7 plants were harvested per compartment. Roots were ignored.

### 2.4 Calculations

#### 2.4.1 Δ*A* and Δ*g*_s_

The magnitude of *A* and *g*_*s*_ responses to step changes in light intensity or *T*_*leaf*_ (Δ*A* and Δ*g*_*s*_, respectively) was calculated as the difference just before the start and at the end of the 15 min of PPFD and/or *T*_*leaf*_ increase. Under the CL + FT protocol, g_s_ showed a WWR, and Δ*g*_*s*_ was therefore calculated as the difference between the maximum *g*_*s*_ reached after *T*_*leaf*_ increased and *g*_*s*_ at the start of the *T*_*leaf*_ increase. The time taken to reach maximum *g*_*s*_ was also calculated.

#### 2.4.2 Slope of *A* and *g*_*s*_

To assess the different phases of photosynthetic induction, the slope of the initial 5 min and final 5 min during the light and/or *T*_*leaf*_ increase was calculated.

#### 2.4.3 Post‐Illumination CO_2_ Burst (PICB)

To calculate the post‐illumination CO_2_ burst (PICB), the first 10 min of *A* after a switch from high to low light intensity was used. We considered that after 10 min the PICB was finished and used the last 30 s average as a reference (*A*_avg_) to calculate integrated *A* with and without a PICB effect. The instantaneous *A*, where the first negative value appeared after it's subtraction with *A*_avg_, was used as a starting point to calculate integrated *A*. To calculate the integrated *A* with a PICB effect, all instantaneous A values were integrated from the starting point until 10 min. Further, to calculate the integrated area without PICB, *A*_avg_ was integrated over the same duration as above. The PICB was calculated as the difference between the two integrated areas.

#### 2.4.4 Statistical Design and Analysis

Eight growth compartments were each divided into two batches of four compartments. Each batch contained two replicate compartments of SQ and AL growth treatments. Seeds for batch 1 and 2 were sown 5‐7 days apart. Thereafter, the whole experiment was repeated in time (batches 3 and 4), bringing the total to eight independent replicate compartments per growth treatment (*n* = 8). Before starting batches 3 and 4, growth treatments were switched between compartments. Per gas exchange measurement protocol, one plant per growth compartment was randomly chosen (*n* = 8 plants per measurement protocol). One randomly selected gas exchange protocol was used on a given day, and three plants (chosen randomly from growth compartments) were measured each day. For AL‐grown plants measured under CL + CT, one replicate was identified as outlier (standardized residuals > 3) and therefore removed from the analysis.

To account for variation in air temperature between the growth compartments (Supporting Information Table S2), the average air temperature during the treatment was introduced as a covariate in the statistical analysis. A linear mixed‐effects model was used with interaction between growth light treatments and measurement cycles of gas exchange measurement protocols as main factors, air temperature during growth as covariate, and random effects of each measured plant nested within the batch. The Lmer function of the LME4 package in R (R version 4.4.2, lme4 1.1–35.5) was used for statistical analysis. The assumptions for normality and homogeneity were assessed using Shapiro‐Wilk's and Levene's test, respectively. Statistically significant differences were assessed at *p* < 0.05. Significant interaction effects were further assessed by pairwise comparisons with Tukey adjustments in the emmeans package within levels of the interacting factors.

## 3 Results

### 3.1 Using an Infrared Lamp to Impose Realistic *T*_*leaf*_ Fluctuations During Gas Exchange Measurements

**Figure 2:**
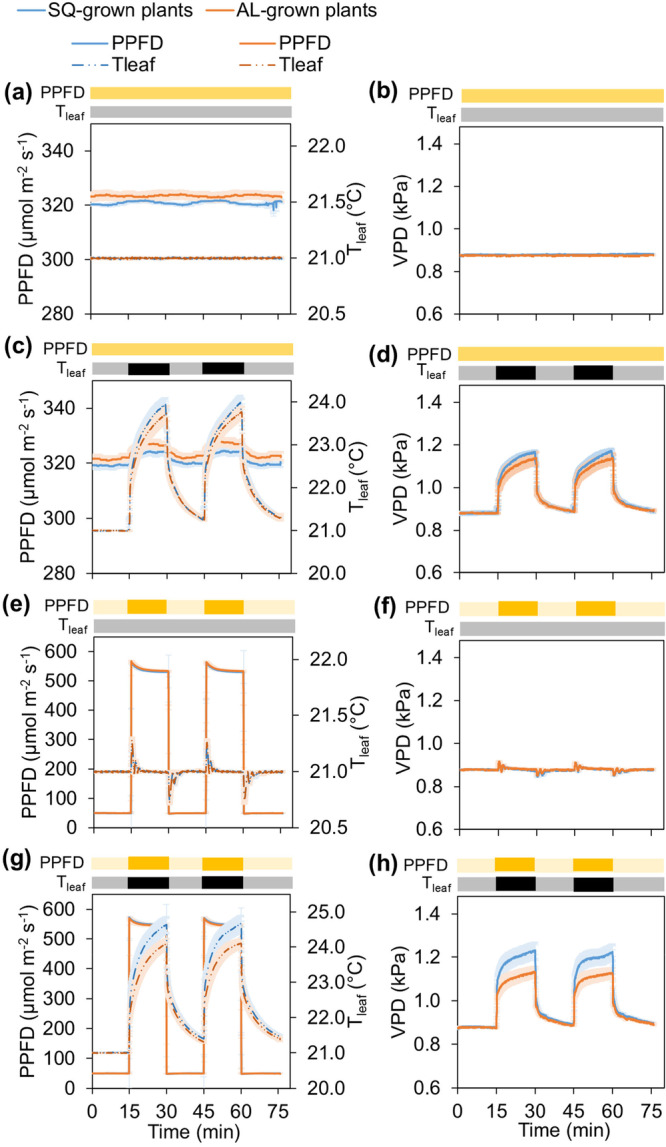
Light intensity (PPFD), leaf temperature (*T*_*leaf*_; left panels), and leaf‐to‐air vapor pressure deficit (VPD; right panels) during light and temperature fluctuations. The protocols were: constant light + constant temperature (CL + CT; a, b), constant light + fluctuating temperature (CL + FT; c, d), fluctuating light + constant temperature (FL + CT; e, f), and fluctuating light + fluctuating temperature (FL + FT; g, h). Yellow and gray bars above panels represent PPFD and *T*_*leaf*_, respectively. Bars with uniform color represent constant PPFD and/or *T*_*leaf*_, bars alternating between light and dark shades indicate fluctuations in PPFD and/or *T*_*leaf*_. Data represent means ± SEM (*n* = 8). [Color figure can be viewed at wileyonlinelibrary.com]

AL‐grown plants showed *T*_*leaf*_ fluctuations within ~2°C in their growing environment, while the temperature of the black tape that was used to quantify the radiative energy load followed the same pattern and was ~2°C–3°C above *T*_*leaf*_ (Supporting Information Figure S3). During CL + FT, step changes in temperature imposed by use of an IR lamp resulted in immediate increases in *T*_*leaf*_ of 3°C–4°C (Figure 2c,g) with relatively small increases in VPD of 0.25–0.35 kPa (Figure 2d,h). Controlling *T*_*leaf*_ at 21°C in FL + CT resulted in mostly stable *T*_*leaf*_ and VPD_leaf_ ( ~ 0.88 kPa), except for brief periods directly after PPFD step changes where *T*_*leaf*_ and VPD changed by ~0.3°C and 0.03 kPa, respectively (Figure 2e,f). Although leaves of both growth treatments received the same amount of IR radiation, SQ‐grown leaves tended to show a greater increase in *T*_*leaf*_ and VPD during CL + FT and FL + FT than AL‐grown leaves, with statistically significant growth treatment effects showing during the FL + FT protocol (*p* = 0.041; Figure 2c,d,g,h).

### 3.2 Increases in *T*_*leaf*_ Cause Transient Uncoupling of *A* and *g*_*s*_ With a Cumulative Effect

**Figure 3:**
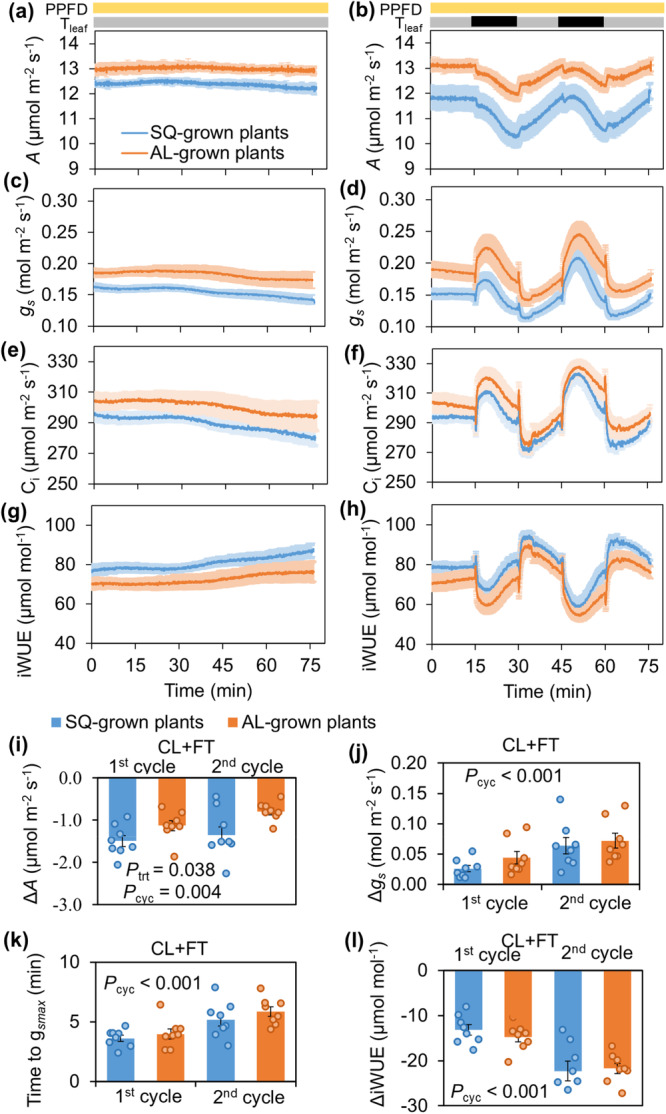
Gas exchange responses of cucumber leaves grown under square wave (SQ) and alternating light (AL) regimes and measured under two protocols: constant light + temperature (CL + CT), and constant light + fluctuating temperature (CL + FT). Temporal responses of *A*, *g*_*s*_, C_i_, and iWUE under CL + CT (a, c, e, g) and CL + FT (b, d, f, h). Changes in *A* (Δ*A*), *g*_*s*_ (Δ*g*_*s*_), iWUE (ΔiWUE), and time to *g*_*smax*,_ under high *T*_*leaf*_ under CL + FT (i–l). Yellow and gray bars above panels represent PPFD and *T*_*leaf*_, respectively. Bars with uniform color represent constant PPFD (320 µmol m^‐2^ s^‐1^) and/or *T*_*leaf*_ (21°C), bars alternating between light and dark shades indicate fluctuations in *T*_*leaf*_ between 21°C and 23.5–24.5°C, respectively. Data represent means ± SEM (*n* = 8). *p*‐values of the main effect of growth treatment (*P*_trt_) and the main effect of measurement cycle (*P*_cyc_) are shown. [Color figure can be viewed at wileyonlinelibrary.com]

AL‐grown leaves showed higher initial steady‐state *A* and *g*_*s*_ than SQ‐grown leaves (Figure 3a–d; Supporting Information S8a,b,d,e). Step increases in *T*_*leaf*_ during CL + FT caused reductions in *A*, which reached a minimum at the end of the high *T*_*leaf*_ phase and then slowly recovered as *T*_*leaf*_ was decreased (Figure 3b). AL‐grown leaves showed less of a reduction in *A* under high *T*_*leaf*_ compared to SQ‐grown leaves, as well as a larger time‐integrated *A* (Figure 3b,i; Supporting Information S8f). The reduction in *A* was smaller in the second cycle of *T*_*leaf*_ increases than during the first cycle (Figure 3b,i). High *T*_*leaf*_ during CL + FT significantly reduced integrated *A* in SQ‐grown leaves (compared to constant *T*_*leaf*_ during CL + CT), but not in AL‐grown leaves (Supporting Information Figure S9a,d).

When *T*_*leaf*_ was rapidly increased during CL + FT, a WWR‐like response in *g*_*s*_ was observed: a rapid increase in *g*_*s*_ in the first few minutes was followed by a slower reduction (Figure 3d). In contrast, stepwise reductions in *T*_*leaf*_ caused a rapid decline in *g*_*s*_, followed by a slow increase (Figure 3d). Additionally, the second cycle of high *T*_*leaf*_ induced a cumulative effect: Δ*g*_*s*_ was significantly larger (Figure 3d,j), and reaching maximum *g*_*s*_ took significantly longer (Figure 3k), during the second than during the first cycle. The time course of C_i_ followed a similar pattern as *g*_*s*_, while iWUE followed an opposite pattern (Figure 3f,h); iWUE was more strongly reduced during the second cycle of high *T*_*leaf*_ (Figure 3l).

### 3.3 Stomatal Pore Aperture Changes With Fluctuating *T*_*leaf*_ Under Constant Light

**Figure 4:**
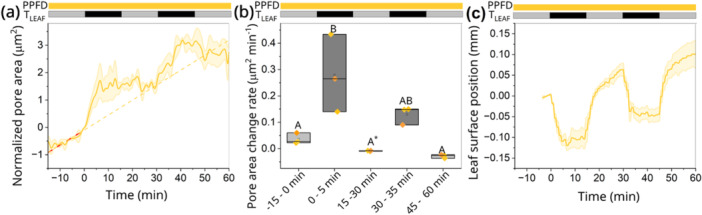
Effect of *T*_*leaf*_ fluctuations on stomatal pore area and leaf surface movement of cucumber leaves grown under an alternating light (AL). (a) Relative change in average stomatal pore area during CL + FT protocol (*n* = 3; Supporting Information Figure S7). Shaded area represents SEM (30 stomata). Pore area was normalized at time t = 0, when individual pore area was 0‐19 µm², with average pore areas of 5, 10, and 11 µm² per replicate plant. Straight line indicates extrapolated fit for initial 15 min prior to first high *T*_*leaf*_ cycle. (b) Rate of pore opening or closing during low (15 min) and high (first 5 min) *T*_*leaf*_ cycles. Boxes and yellow dots represent values from individual plants, open squares indicate means. Letters denote statistically significant differences based on repeated‐measures ANOVA and Tukey post‐hoc test. Asterisk indicates that slopes were not significantly different from zero. (c) Leaf surface movement relative to its position at t = 0. Relative position of leaf surface based on autofocus of microscope: negative values indicate closer proximity of leaf surface to microscope. Yellow and gray bars above panels represent PPFD and *T*_*leaf*_, respectively. Bars with uniform color represent constant PPFD (730–740 µmol m^‐2^ s^‐1^), bars alternating between light and dark shades indicate fluctuations in *T*_*leaf*_ between 26°C and 27°C and 32.5–33.5°C, respectively. [Color figure can be viewed at wileyonlinelibrary.com]

Abaxial stomata of AL‐grown leaves showed a slight opening trend at the start of the CL + FT protocol, despite initial exposure to constant conditions (time = 0 min in Figure 4a). Step increases in *T*_*leaf*_ increased the average stomatal opening trend compared to the period without heating (Figure 4a,b) similar to the initial WWR‐like response of *g*_*s*_ (Figure 3d). In contrast to the gas exchange response, however, stomatal aperture did not decrease after reaching a maximum under high *T*_*leaf*_ (Figure 4a). A trend towards reductions in pore aperture was only observed in the last period without extra thermal irradiance (Figure 4b). Stomatal opening during the entire CL + FT was larger than expected based on extrapolations from the initial opening trend, indicating an accumulative effect of the thermal irradiance (Figure 4a,b). Leaf surface movements (Figure 4c) indicated bending of the leaf surface during CL + FT: the leaf surface moved towards the microscope after *T*_*leaf*_ increased (i.e., after the IR lamp was switched on), and away from the microscope when *T*_*leaf*_ was reduced.

### 3.4 Concurrent Increases in *T*_*leaf*_ and Light Intensity Enhanced the Magnitude and Rapidity of *A* and *g*_*s*_

**Figure 5:**
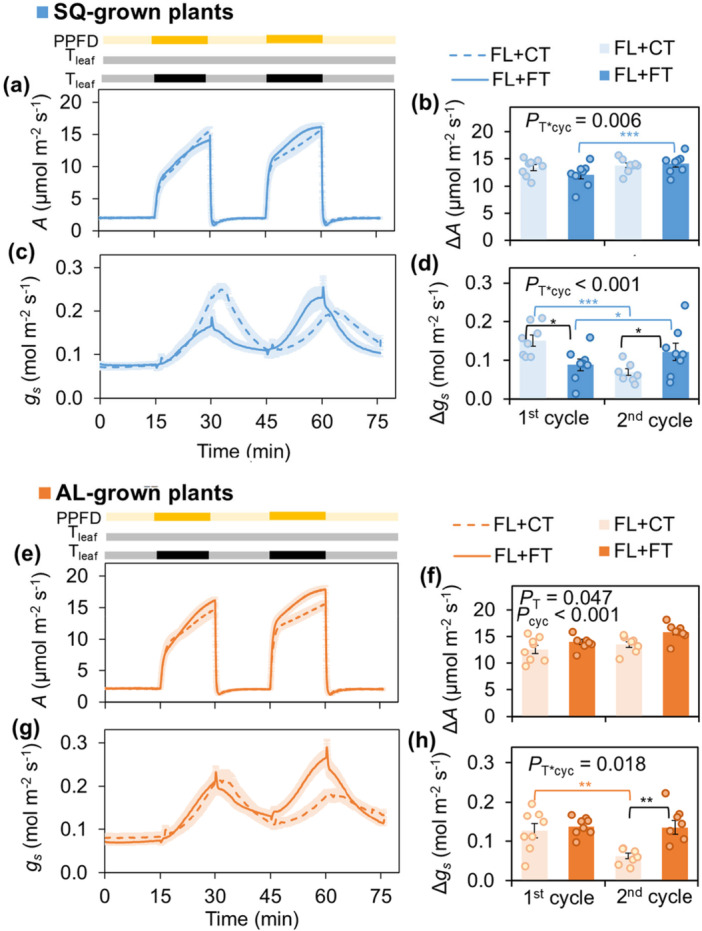
Effects of temperature on gas exchange responses of cucumber leaves grown under square wave (SQ; a–d) and alternating light (AL, e–h) measured under fluctuating light + constant temperature (FL + CT) and fluctuating light + fluctuating temperature (FL + FT). Every 15 min, leaves were exposed to either simultaneous changes in leaf temperature (FT; between 21°C and 24.5°C) and FL (between 50 and 550 μmol m^‐2^ s^‐1^), or FL under constant temperature (CT, 21°C). Temporal responses of *A* and *g*_*s*_ in SQ (a, c) and AL‐grown leaves (e, g). Changes in *A* (Δ*A*) and *g*_*s*_ (Δ*g*_*s*_) in SQ (b, d) and AL‐grown leaves (f, h). Yellow and gray bars above panels represent PPFD and *T*_*leaf*_, respectively. Bars with uniform color represent constant *T*_*leaf*_, bars alternating between light and dark shades indicate fluctuations in PPFD and/or *T*_*leaf*_. Data represent means ± SEM (*n* = 8). *p*‐values of the main effect of temperature (*P*_T_), measurement cycle (*P*_cyc_), and the interaction effect between temperature and measurement cycle (P_T*cyc_) are shown. Asterisks indicate the significant pairwise comparisons within the levels of the interacting factors: * *p* < 0.05, ** *p* < 0.01, and *** *p* < 0.001. [Color figure can be viewed at wileyonlinelibrary.com]

**Figure 6:**
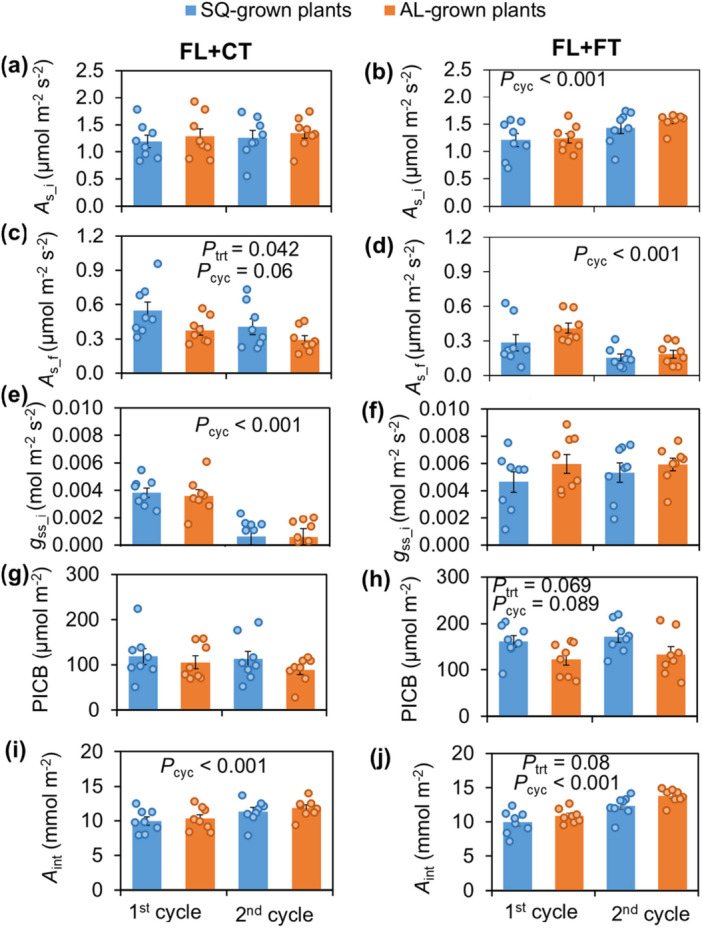
Rate of increase in *A* and *g*_*s*_ as estimated by slope, post‐illumination CO_2_ burst, and integrated *A* measured under fluctuating light + constant temperature (FL + CT; a, c, e, g, i) and fluctuating light + fluctuating temperature (FL + FT; b, d, f, h, j). Cucumber plants were grown under square wave (SQ) or alternating light (AL). Slope of the initial 5 min after PPFD and/or *T*_*leaf*_ increase for *A* (*A*_s_i_; a, b) and *g*_*s*_ (*g*_*s*s_i_; e, f), slope of the final 5 min before PPFD and/or *T*_*leaf*_ decrease for *A* (*A*_s_f_; c, d). Post‐illumination CO_2_ burst (PICB) after PPFD and/or *T*_*leaf*_ decrease for FL + CT (g) and FL + FT (h). Time‐integrated rate of photosynthesis (*A*_int_) during the respective cycles of PPFD and/or *T*_*leaf*_ increase for FL + CT (i) and FL + FT (j). Data represent means ± SEM (*n* = 8). *p*‐values of the main effect of growth treatment (*P*_trt_) and the main effect of measurement cycle (*P*_cyc_) are shown. [Color figure can be viewed at wileyonlinelibrary.com]

Upon an increase in PPFD (at constant *T*_*leaf*_; FL + CT), *A* showed a biphasic response: a rapid initial increase and a slower secondary increase (Figure 5a,e; Supporting Information S10a). The magnitude of increase in *A* under high PPFD during FL + CT was significantly different in the second than the first cycle with an increase of 0.7 ± 0.24 µmol m^−2^ s^−1^ (Supporting Information Figure S10i), resulting in significantly larger time‐integrated *A* during the second cycle for both growth treatments (Figure 6i). Notably, during the second cycle of high PPFD during FL + CT, *g*_*s*_ increased much less (Figure 5c,g; Supporting Information S10b,j), and with a much smaller initial slope (Figure 6e), than during the first cycle. Due to the rapid increase in *A* after the step increase in PPFD during FL + CT, C_i_ quickly dropped to a minimum, followed by a slow increase due to stomatal opening until the end of the high PPFD phase (Supporting Information Figure S10c). iWUE rapidly increased to a maximum directly after the PPFD increase and then slowly declined until the end of the PPFD increase (Supporting Information Figure S10d). There were no notable differences between SQ and AL‐ grown leaves under FL + CT (Supporting Information Figure S10a,b,i,j).

Under FL + FT, when both *T*_*leaf*_ and PPFD were changed simultaneously, *g*_*s*_ and to a lesser extent *A* showed marked differences compared to FL + CT (Figure 5): for example, the magnitude and rapidity of the *g*_*s*_ increase were significantly larger in the second cycle of high *T*_*leaf*_ and PPFD, compared to the response at FL + CT (Figure 5c,d,g,h; Supporting Information S10b,f). While *A* still showed a linear increase in the FL + CT protocol, *A* seemed to plateau sooner as quantified by the smaller slope of the last 5 min during PPFD and/or *T*_*leaf*_ increases, especially during the second cycle of FL + FT (Figure 5a,e; Supporting Information S11c,g). Compared to the first cycle of high *T*_*leaf*_ and PPFD during FL + FT, the magnitude and rate of increase of *A* were significantly higher in the second cycle (Figure 5b,f; 6b; Supporting Information S10k). AL‐grown leaves showed a significantly stronger increase in *A* than SQ‐grown leaves under high *T*_*leaf*_ and PPFD during FL + FT (Supporting Information Figure S10k). Time‐integrated *A* during the second cycle of high *T*_*leaf*_ and PPFD during FL + FT was larger than that under FL + CT, and AL‐grown leaves tended to have larger time‐integrated *A* than SQ‐grown leaves (Figure 6j). Concurrent *T*_*leaf*_ and PPFD reductions during FL + FT enhanced the PICB compared to FL + CT in SQ‐grown leaves (Supporting Information Figure S11d). In AL‐grown leaves, PICB under FL + FT tended to be larger than under FL + CT in the second cycle compared to the first cycle (Supporting Information Figure S11h). Furthermore, AL‐grown leaves tended (*p* = 0.07) to have a smaller PICB under FL + FT than SQ‐grown leaves (Figure 6h).

### 3.5 Reductions in *A* Due to Step Increases in *T*_*leaf*_ at Ambient Air Disappeared at Low O_2_

**Figure 7:**
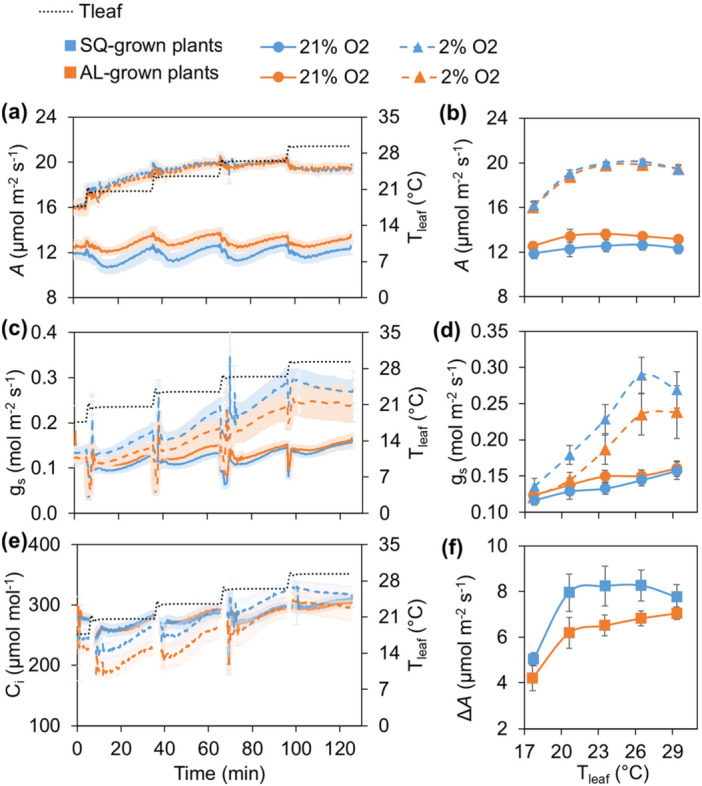
Temperature response of leaf gas exchange at 21% and 2% O_2_ of cucumber leaves grown under square‐wave (SQ) and alternating light (AL) regimes. Temporal responses of *A*, *g*_*s*_, and C_i_ after step increases in *T*_*leaf*_ between 18°C and 30°C, at steps of 3°C applied every 30 min (a, c, e). Temperature response curves of *A* and *g*_*s*_ (b, d). Temperature response of the difference between *A* at 21% and 2% O_2_ (Δ*A*; f). Data represent means ± SEM (*n* = 8 at 21% O_2_ and *n* = 4 at 2% O_2_). [Color figure can be viewed at wileyonlinelibrary.com]

Based on our finding that *A* declined upon step increases in *T*_*leaf*_ during CL + FT (Figure 3b), we investigated whether *A* recovered if *T*_*leaf*_ was increased for longer, and further explored the underlying mechanism for this decline. The temperature responses of *A* and *g*_*s*_ were measured close to the average growth PPFD (300 μmol m^−2^ s^−1^), and at ambient and low O_2_ concentrations. At 21% O_2_, *A* declined directly after a step increase in temperature and then recovered (Figure 7a). This decline in *A* disappeared at 2% O_2_ (Figure 7a). Under 2% O_2_, *A* increased right after step increases in temperature until 27°C, and then slightly declined between 27°C and 30°C in leaves from both growth treatments (Figure 7a,b). As expected, steady‐state *A* was substantially higher at 2% O_2_ compared to 21% O_2_ (Figure 7b). While steady‐state *A* and C_i_ tended to be stable over the temperature range from 18°C to 30°C at 21% O_2_, *g*_*s*_ increased near‐linearly with temperature (Figure 7c). In contrast to 21% O_2_, there was a pronounced linear increase in *g*_*s*_ at 2% O_2_ until 27°C (Figure 7d). No statistically significant differences were observed between growth treatments (Figure 7). In contrast to the transient g_s_ response under the CL + FT protocol (Figure 3d), step increases in temperature in the response curve did not induce WWR of g_s_ (Figure 7c), likely due to the slower increase in temperature.

### 3.6 Alternating Light Intensity Affects Plant Biomass and Stomatal Density

**Figure 8:**
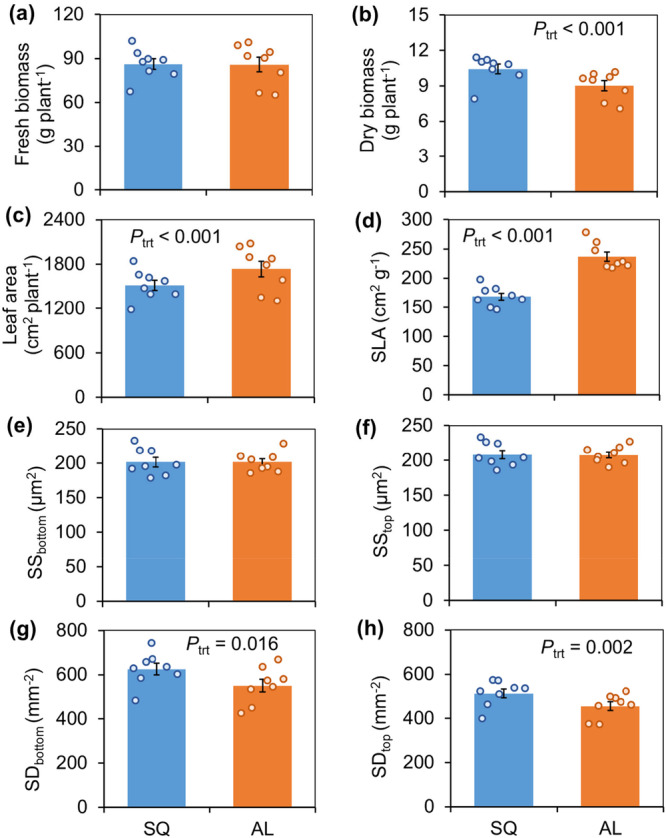
Plant growth and morphological traits of stomata: (a) fresh above‐ground biomass, (b) dry above‐ground biomass, (c) leaf area, (d) specific leaf area (SLA), (e) stomatal size on bottom leaf surface (SS_bottom_), (f) stomatal size on top leaf surface (SS_top_), (g) stomatal density on bottom leaf surface (SD_bottom_), (h) stomatal density on top leaf surface (SD_top_). Cucumber plants were grown under square wave (SQ) and alternating light (AL) for 26–27 days. Data represent means ± SEM (*n* = 8). *p*‐value of the main effect of growth treatment (*P*_trt_) is shown. [Color figure can be viewed at wileyonlinelibrary.com]

Above‐ground dry biomass was significantly lower in AL compared to SQ‐grown plants, while fresh above‐ground biomass was not significantly different (Figure 8a,b). AL‐grown plants displayed significantly larger leaf area and SLA than SQ‐grown plants (Figure 8c,d). While stomatal size on both leaf surfaces was unaffected by growth treatments, stomatal density was significantly lower in AL than in SQ‐grown plants on both leaf surfaces (Figure 8e–h).

## 4 Discussion

Despite their abundance in nature, the effects of concurrent fluctuations in light intensity and leaf temperature (*T*_*leaf*_) on photosynthetic gas exchange are not well explored. Our results show that not only the magnitude (as previously described), but the rapidity of temperature change strongly shapes leaf carbon and water balance. Moderate yet rapid increases in *T*_*leaf*_ (~3°C) at constant light (CL + FT) caused large stomatal conductance (*g*_*s*_) increases (up to ~40%), as well as decreases in *A* ( ~ 11%), revealing that leaves are highly sensitive to fast thermal perturbations (Figure 3b,d,i,j). When combined with fluctuating light, this rapid *T*_*leaf*_ increase caused faster and stronger responses of *g*_*s*_ and *A*, with a cumulative effect for each new *T*_*leaf*_ increase (Figure 5; Supporting Information S11b,c,f). These findings highlight that realistic rapid temperature dynamics can fundamentally alter how leaves respond to sunflecks and should be considered when interpreting plant function under fluctuating environmental conditions.

### 4.1 Stomatal Responses to Rapid *T*_*leaf*_ Increases Exert a Mechanical Advantage Under Sunflecks

The first rapid yet moderate increases in *T*_*leaf*_ during CL + FT induced a small increase in VPD ( ~ 0.28 kPa; Figure 2d) but a 24% transient increase in *g*_*s*_ (Figure 3d,j). Similarly to what occurred during a WWR, this was likely caused by the altered water status of epidermal cells which was faster than guard cells can adjust. The resultant drop in epidermal turgor likely passively enabled wider stomatal aperture, which after ~4 min became actively coordinated again with the variations in *A*. It is interesting to note that after *T*_*leaf*_ decreased, a rapid decrease in *g*_*s*_ followed by a slow increase was observed (Figure 3d), representing the inverse of the WWR. This pattern suggested a rapid increase in epidermal cell turgor due to the sudden reduction in transpiration constraining stomatal aperture. These two types of WWR are compatible with previous work on the mechanical advantage (J. Robert Cooke et al. 1976; Meidner and Bannister 1979; Franks et al. 1998; Franks and Farquhar 2007) and may occur frequently during sunflecks and leaf fluttering (Roden and Pearcy 1993; Leakey et al. 2003; Fauset et al. 2018).

Microscopy confirmed the general response pattern of *g*_*s*_ in response to rapid *T*_*leaf*_ increase during CL + FT (Figure 4a,b), although with some discrepancies (Figure 3d). These discrepancies likely arose from methodological differences: for example, microscopy was performed on the abaxial side only, while gas exchange represented both sides of the leaf. The abaxial stomata only received indirect light that was reflected or passing through the leaf, potentially leading to reduced response compared to the adaxial stomata (Wall et al. 2022). In addition, the lower boundary layer conductance under the microscope than inside the gas exchange cuvette likely altered *T*_*leaf*_ and VPD variations.

Importantly, microscopy revealed leaf movements showing a consistent response to rapid *T*_*leaf*_ increases. These movements were likely driven by changes in epidermal cell turgor over the leaf surface further supporting the WWR hypothesis. Additionally, we observed a cumulative increase in *g*_*s*_ (40%, Figure 3d,j) in both gas exchange and microscopy data (Figure 4a,b) during the second rapid increase in *T*_*leaf*_ under CL + FT, which were concomitant with the cumulative increase in the position of the leaf surface. We hypothesized that successive reductions in epidermal cell turgor in response to *T*_*leaf*_ were not fully restored, which over time helped sustain a wider stomatal aperture.

The role of epidermal cell turgor on the dynamic regulation of stomatal aperture to environmental cues is not well described nor modeled. Epidermal cell turgor is often assumed constant and studies have focused only on the first aspect of the competition between guard and epidermal cell turgor, i.e. how guard cells need to overcome epidermal turgor pressure (Robert Cooke et al. 1976; Edwards et al. 1976; Meidner and Bannister 1979; Jezek et al. 2019; Cong et al. 2024), not the other way around. Although we did not measure turgor pressures of guard and epidermal cells, we observed a general change in leaf turgor, as shown by leaf surface movements during *T*_*leaf*_ changes (Figure 4c), which had previously been observed under large changes in light intensity (van den Berg et al. 2025). This dynamic leaf turgor change suggested that we need to revisit the exact contribution of epidermal cell turgor pressures to dynamic variation in *g*_*s*_.

In terms of stomatal rapidity, the expected response to two successive light intensity changes was a slower *g*_*s*_ increase during the second high light exposure, which was indeed observed here (Figure 5c,g; Supporting Information S10b). Storage and release of ions such as Ca^2+^ (Jezek et al. 2021) or K^+^ (Hosy et al. 2003; Lebaudy et al. 2008) can explain the slower stomatal response, as successive response can reduce their availability. However, a simultaneous increase of *T*_*leaf*_ with light intensity during FL + FT caused a more rapid and larger change in *g*_*s*_ compared to that at FL + CT (Figure 5c,g; S11b,f) suggesting that a temperature increase facilitates the stomatal response to FL. It is in line with previous work showing faster *g*_*s*_ responses in species in which epidermal turgor pressure was lowered by higher VPD (Pichaco et al. 2024). Overall, it supports our hypothesis of a varying mechanical advantage in response to temperature fluctuations, facilitating stomatal opening responses in the light.

### 4.2 Rapid *T*_*leaf*_ Fluctuations Interact With Photorespiratory Processes Under Sunflecks

After a rapid increase of *T*_*leaf*_ under CL + FT, *A* became uncoupled from *g*_*s*_ and declined up to 13% after ~15 min (Figure 3b,d), indicating that biochemical rather than diffusional limitations dominated during this period. The disappearance of this decline under low O₂ (Figure 7a) suggested that the transient decrease in *A* arose from a short‐term increase in photorespiratory flux, likely driven by a temporary imbalance in photorespiratory metabolite pools (Fu and Walker 2023). As temperature increases, the proportion of potential carbon uptake that is lost to photorespiration should increase (Long 1991), yet our results showed it was stable in the 20°C–26°C range (Figure 7f). A likely explanation for the transient response would be a transient increase in RuBP availability before metabolic pools re‐equilibrate, although we cannot identify the exact causes. This re‐equilibration took ~30 min and likely impacted photosynthesis induction when *T*_*leaf*_ and light were changed simultaneously, although it is not identifiable from the kinetics.

In contrary to Pankasem et al. (2024), which observed rapid equilibrium in *A* in Arabidopsis, when transitioning from 20°C to 23°C, *A* decreased transiently in cucumber plants and recovered after 30 min (Figure 7a). This suggested that rapid *T*_*leaf*_ increases induced transient responses that are different from the expected steady state variations and may be species specific. The smaller decrease in *A* after the second *T*_*leaf*_ increase suggested that part of the photorespiratory or biochemical machinery had partially adjusted to the first thermal perturbation, reducing the magnitude of the transient imbalance. Together, these results indicate that rapid *T*_*leaf*_ increases induce short‐lived metabolic disruptions distinct from steady‐state temperature responses.

### 4.3 Rapid *T*_*leaf*_ Fluctuations Interact With Photosynthetic Processes Under Sunflecks

Up to an optimum, increased *T*_*leaf*_ during a sunfleck has been shown to promote enzyme activation in the RuBP regeneration pathway, activation of Rubisco, and stomatal opening (Kaiser et al. 2015, 2017). Here, photosynthesis induction was faster and larger under FL + FT compared to FL + CT, especially during the second response, suggesting that the increase in *T*_*leaf*_ resulted in faster biochemical reactions. This was especially visible during the second response (Figure 5a,e; Supporting Information S11a,e) and was probably helped by increased *g*_*s*_ (Figure 5c,g; Supporting Information S11b,f). Similarly to our results, Kang et al. (2020) also observed that *A* reached steady‐state faster under concurrent increases of *T*_*leaf*_ with light intensity compared to constant *T*_*leaf*_.

After light intensity was reduced, we observed a post‐illumination CO_2_ burst (PICB), which has been proposed to be proportional to photorespiration (Vines et al. 1983). The PICB was enhanced when both *T*_*leaf*_ and light intensity during FL + FT were decreased simultaneously (Supporting Information Figure S11d,h), however it occurred in an opposite direction compared to CL + FT (Figure 3b), suggesting different mechanisms. While the faster and larger *A* increase can be beneficial for photosynthetic induction during sunflecks, a high PICB can reduce carbon gain attributed to a sunfleck. Our results highlight the role of photorespiration at different moments in the response to sunflecks, and call for an inclusion of the effect of *T*_*leaf*_ on photorespiration dynamics in dynamic leaf photosynthesis models (Kirschbaum et al. 1997; Pearcy et al. 1997; Morales et al. 2018). Previous models described transient imbalance in metabolic fluxes when light intensity changed by implementing metabolic pools of the Calvin cycle with distinct rate constants. These models simulate the kinetic lag between intermediates and adding temperature responses of enzyme activities with an Arrhenius type equation would reveal emerging properties when temperature is changed, resulting from transient imbalances in metabolic pools.

### 4.4 Acclimation to Growth Under Fluctuating Light Intensity

As previously demonstrated by Shrestha et al. (2025), cucumber plants grown under a fluctuating light regime acclimated by increasing their leaf area with less dense leaves and lower SD, but higher *A* and *g*_*s*_ (Figure 3a–d; 8c,d,g,h). An overall reduction of photorespiration in AL‐grown leaves and therefore, a lower reduction in *A* following an increase in *T*_*leaf*_ contributed to higher integrated carbon gain (Figure 3b,i;7f; Supporting Information S8f). This suggested that growth under AL entails acclimatory adjustments in photorespiratory mechanisms, providing them with an advantage under *T*_*leaf*_ fluctuations. This was also supported by AL‐grown leaves potentially having higher integrated *A* because of significantly larger increase in *A* and potentially lower PICB when both light and *T*_*leaf*_ changed under FL + FT, which can also increase carbon gain, compared to SQ‐grown plants (Figure 6h; Supporting Information S8l). Although relatively small, these acclimatory adjustments due to growth under AL could give a slight advantage in terms of carbon gain under sunflecks wherein *T*_*leaf*_ varies concurrently with light. In general, the response of *g*_*s*_ to temperature did not show a large effect size between different growth conditions, suggesting that the WWR did not acclimate.

### 4.5 Temperature Variations Under Growth Conditions and in the Gas Exchange Cuvette

The magnitude of *T*_*leaf*_ fluctuations in response to light intensity changes in AL grown leaves was larger than that occurring in the gas exchange cuvette when using default settings (Supporting Information Figure S3; S4). This was the result of different leaf energy balances, with the difference in boundary layer conductance and cuvette wall temperature being important contributing factors. The mismatch between steady‐state natural and leaf cuvette temperature conditions has been reported previously (Still et al. 2019), and here we stress the importance of realistic rapid temperature variations to understand stomatal behavior. For example, WWR‐like responses due to *T*_*leaf*_ increases were observed only in the measurement set‐up that used an infrared lamp, but not when using the gas exchange cuvette built‐in temperature controls, as this was too slow to induce WWR‐like responses (Figures 3d and 7c). The stomatal responses observed here have therefore been overlooked due to technical limitations preventing to recreate the exact conditions occurring in nature.

## 5 Conclusions

Using a novel measurement set‐up, we found that concurrent and rapid increases in leaf temperature and light intensity accelerate stomatal opening and photosynthetic induction compared with light increases alone. Microscopy confirmed these responses and revealed temperature‐dependent changes in epidermal tissues, supporting a biomechanical contribution to enhanced stomatal opening. In nature, leaves may experience many light and leaf temperature fluctuations daily, and the cumulative effect observed here suggests that this will lead to different outcomes based on the history of these fluctuations. Rapid increases in leaf temperature alone triggered a transient wrong‐way–like stomatal response and a temporary reduction in *A*, likely driven in part by enhanced photorespiration. The modest temperature change ( ~ 3°C) that triggered these responses suggests that such phenomena may occur more frequently and have a greater impact than previously understood under natural conditions. Collectively, our findings highlight temperature dynamics as a critical but underappreciated driver of stomatal behavior and photosynthetic performance, underscoring the need to incorporate realistic thermal fluctuations into experimental and modeling frameworks.

## Conflicts of Interest

The authors declare no conflicts of interest.

## Supporting information


**Table S1:** Light and temperature settings used for the gas exchange and microscopy measurement protocols. **Table S2:** Average temperature per growing compartment (duration: 21 days) used as covariate in the statistical model.

## Data Availability

Available upon request.
