# Supplementary material for: Concurrent Temperature and Light Intensity Fluctuations Promote Stomatal Opening and Non‐Steady State Photosynthesis
Source: Plant Cell Environ. 2026 Apr 2;49(8):5005–21. doi: 10.1111/pce.70509 (PMC13353751; doi:10.1111/pce.70509)
Supplement: Supplementary file 1 — Table S1: Light and temperature settings used for the gas exchange and microscopy measurement protocols. Table S2: Average temperature per growing compartment (duration: 21 days) used as covariate in the statistical model. [file PCE-49-5005-s001.docx]

| Supporting information   \| Table S1. Light and temperature settings used for the gas exchange and microscopy measurement protocols \| \| \| \| --- \| --- \| --- \| \| Measurement protocol \| Light intensity (µmol m^-2^ s^-1^) \| Leaf temperature (°C) \| \| Gas exchange measurements \|  \|  \| \| Constant light (CL) + Constant temperature (CT) \| 320 \| 21 \| \| Constant light (CL) + Fluctuating temperature (FT) \| 320 \| 21 🡺 23.5-24.5 \| \| Fluctuating light (FL) + Constant temperature (CT) \| 50 🡺 550 \| 21 \| \| Fluctuating light (FL) + Fluctuating temperature (FT) \| 50 🡺 550 \| 21 🡺 23.5-24.5 \| \| Microscopy measurements \|  \|  \| \| Constant light (CL) + Fluctuating temperature (FT) \| 730-740 \| 26-27 🡺 32.5-33.5 \|   Table S2. Average temperature per growing compartment (duration: 21 days) used as covariate in the statistical model | | | | |
| --- | --- | --- | --- | --- | --- | --- | --- | --- | --- | --- | --- | --- | --- | --- | --- | --- | --- | --- | --- | --- | --- | --- | --- | --- | --- | --- | --- | --- | --- | --- | --- |
| Batch number | Compartment | Growth treatment | Repetition | Air temperature (°C) |
| 1 | 1 | CL | 1 | 21.6 |
| 1 | 2 | CL | 2 | 21.5 |
| 1 | 3 | FL | 1 | 21.4 |
| 1 | 4 | FL | 2 | 21.2 |
| 2 | 5 | CL | 3 | 21.7 |
| 2 | 6 | CL | 4 | 21.1 |
| 2 | 7 | FL | 3 | 22.1 |
| 2 | 8 | FL | 4 | 20.8 |
| 3 | 3 | CL | 5 | 21.6 |
| 3 | 4 | CL | 6 | 21.3 |
| 3 | 2 | FL | 6 | 21.7 |
| 3 | 1 | FL | 5 | 21.7 |
| 4 | 7 | CL | 7 | 22.2 |
| 4 | 8 | CL | 8 | 20.8 |
| 4 | 5 | FL | 7 | 21.6 |
| 4 | 6 | FL | 8 | 20.9 |


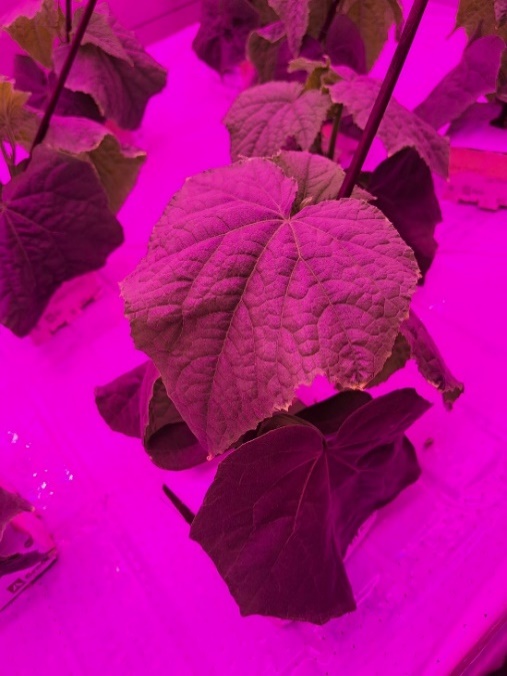


Fig. S1 Cucumber plant tilted to one side, using a wooden stick. This was done to make sure that the majority of leaf #4 was exposed to treatment light.


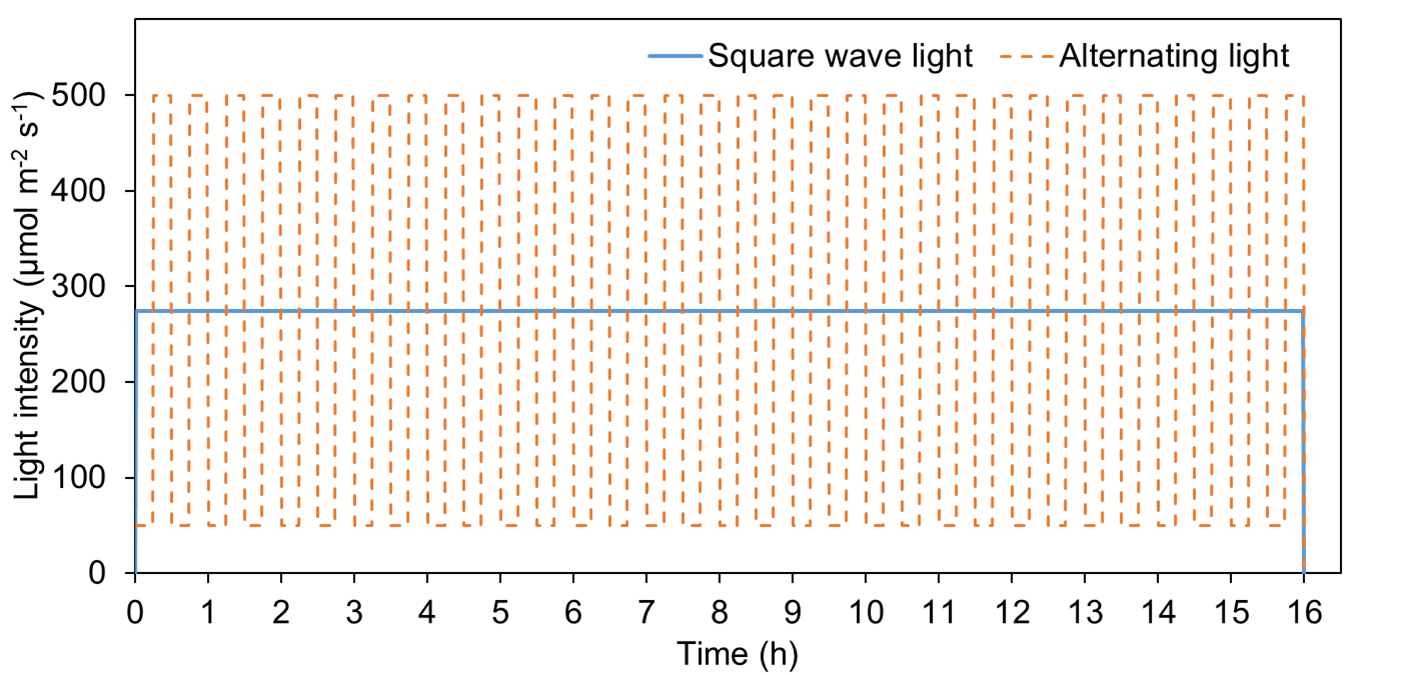


Fig. S2 Light regimes used for growing cucumber plants in the climate container.


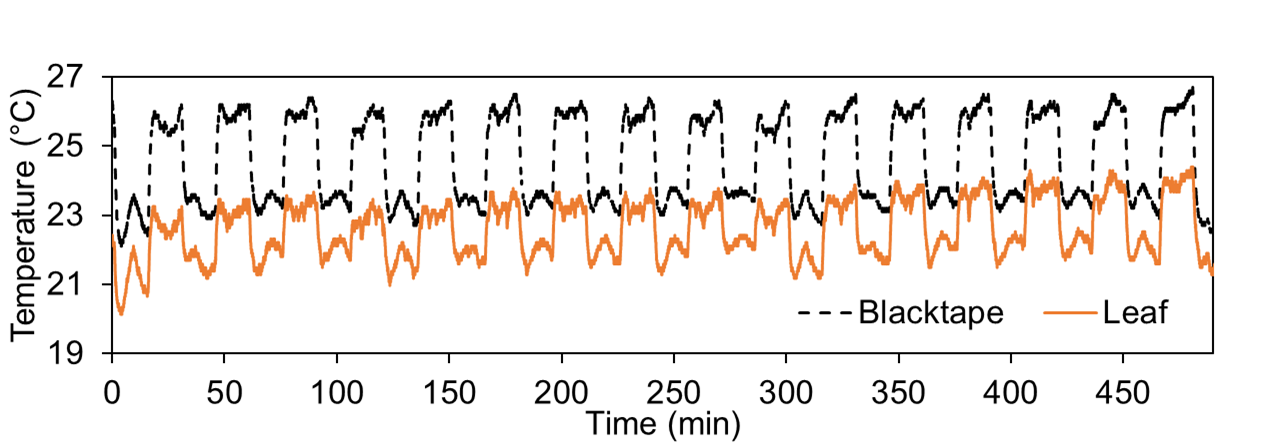


Fig. S3 Temperature fluctuations of leaf and black tape under the growth alternating light (AL) treatment.


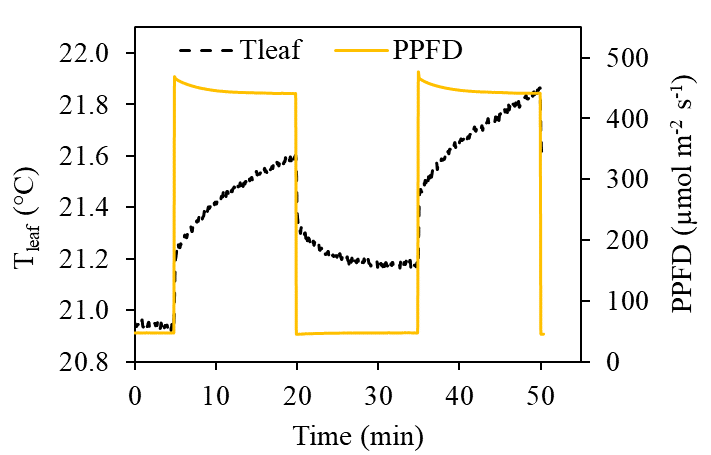


Fig. S4 Leaf temperature of leaf clamped inside the cuvette of the Li-6800 exposed to fluctuating light (FL).


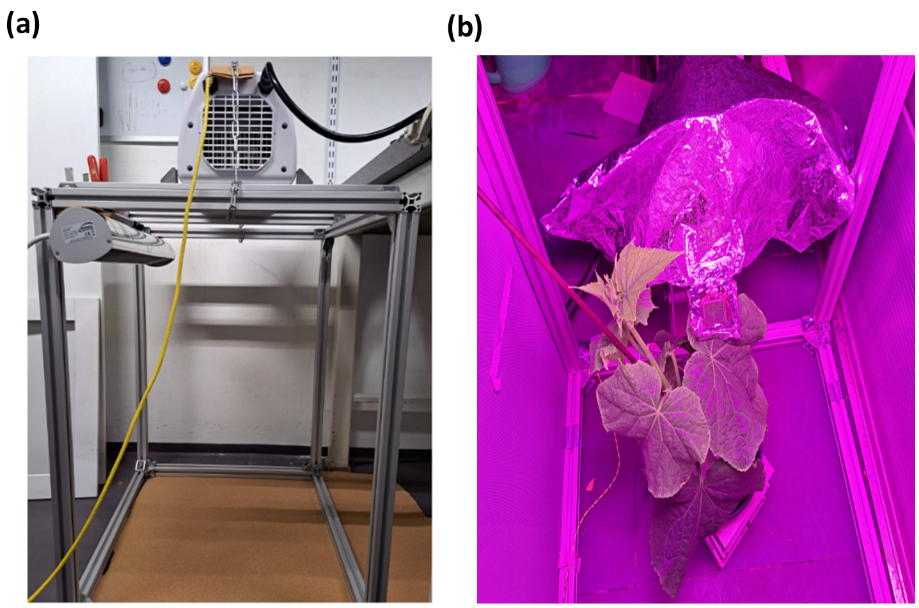


Fig. S5 Metal frame set-up for gas exchange measurement protocols (a) and measurement inside the metal frame using a clear top cuvette, with the rest of Li-6800 covered in a reflective plastic to avoid heating of the machine by the infrared lamp (b).


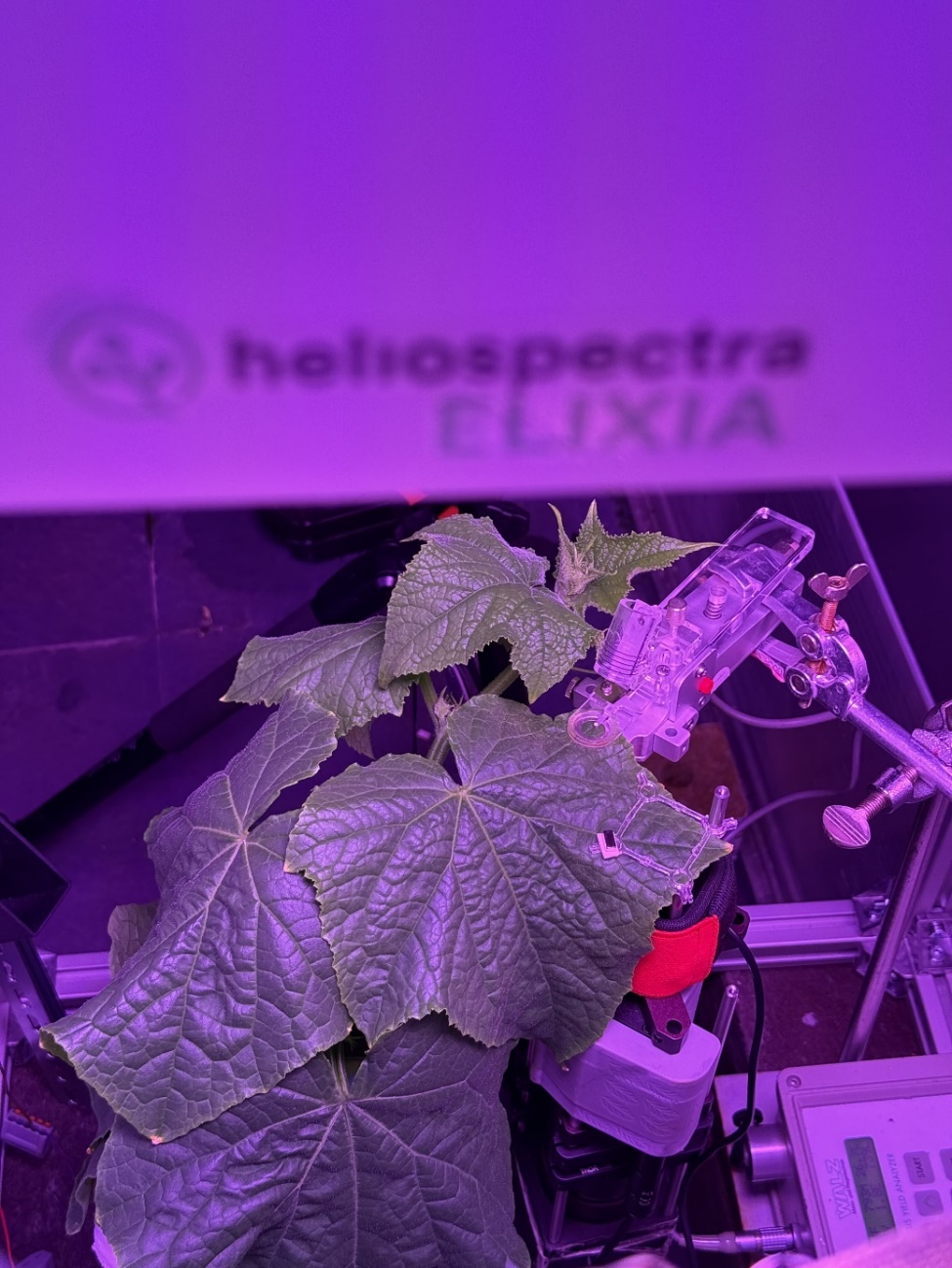


Fig. S6 Microscopy measurement setup inside the metal frame using the custom microscope and the leaf clip from a mini-PAM for PAR and T_leaf_ measurements.


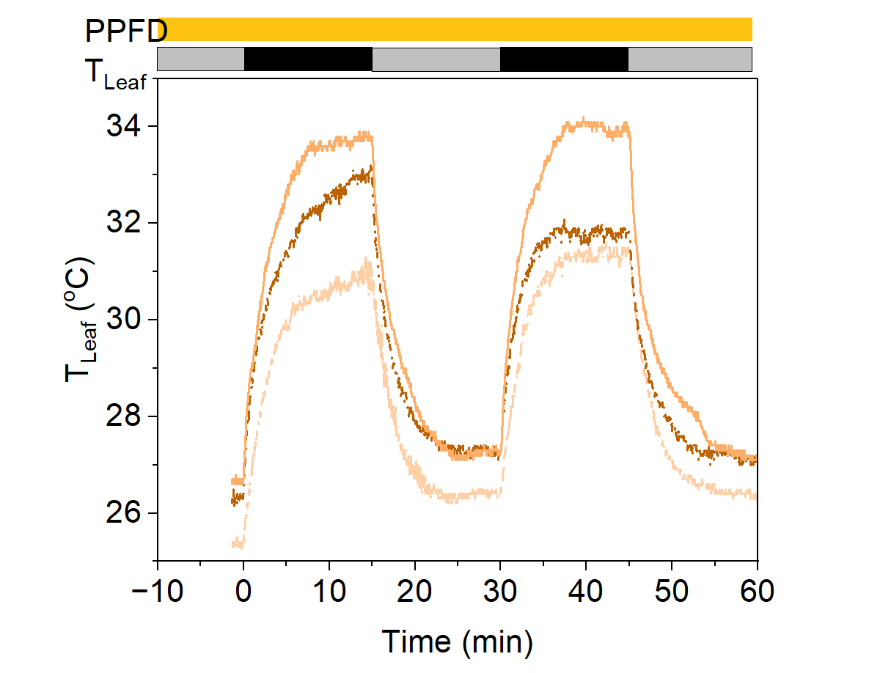


Fig. S7 Leaf Temperature changes during the constant light +constant temperature (CL+FT) protocol with the microscope. Each plant response is plotted.


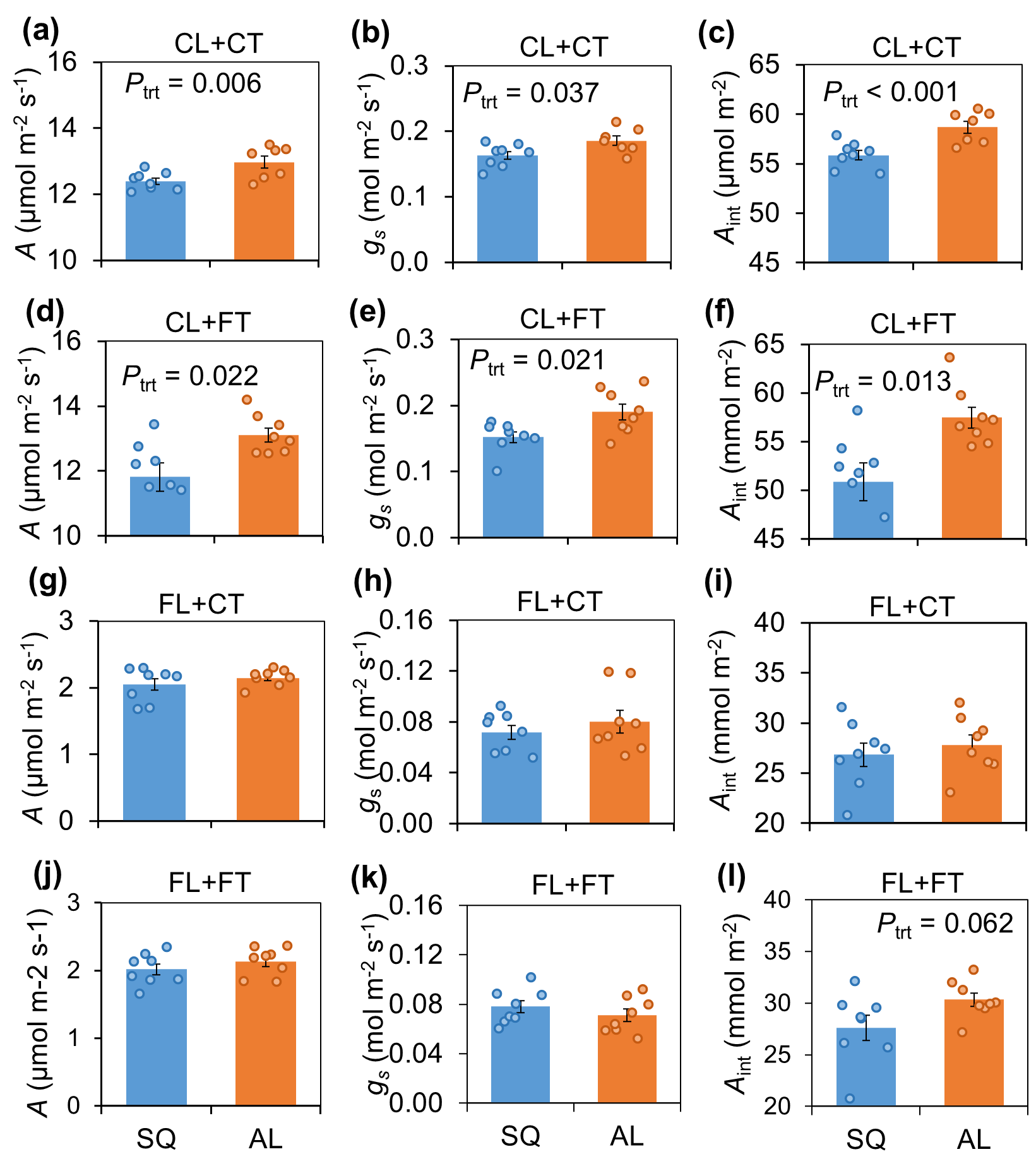


Fig. S8 Steady-state *A* and g_s_ of the four measurement protocols calculated as an average of 30 s data at the beginning of each protocol, and integrated *A* (*A*_int_) over the whole duration of the measurement protocols. Cucumber plants grown under square wave (SQ) and alternating light (AL) were measured under four gas exchange protocols: constant light + constant temperature (CL+CT), constant light + fluctuating temperature (CL+FT), fluctuating light + constant temperature (FL+CT), and fluctuating light + fluctuating temperature (FL+FT). Steady-state *A*, g_s_, and *A*_int_ for CL+CT (a-c), CL+FT (d-f), FL+CT (g-i), and FL+FT (j-l). Data represent means ± SEM (n=8). *P*-value of the main effect of growth treatment (*P*_trt_) is shown.


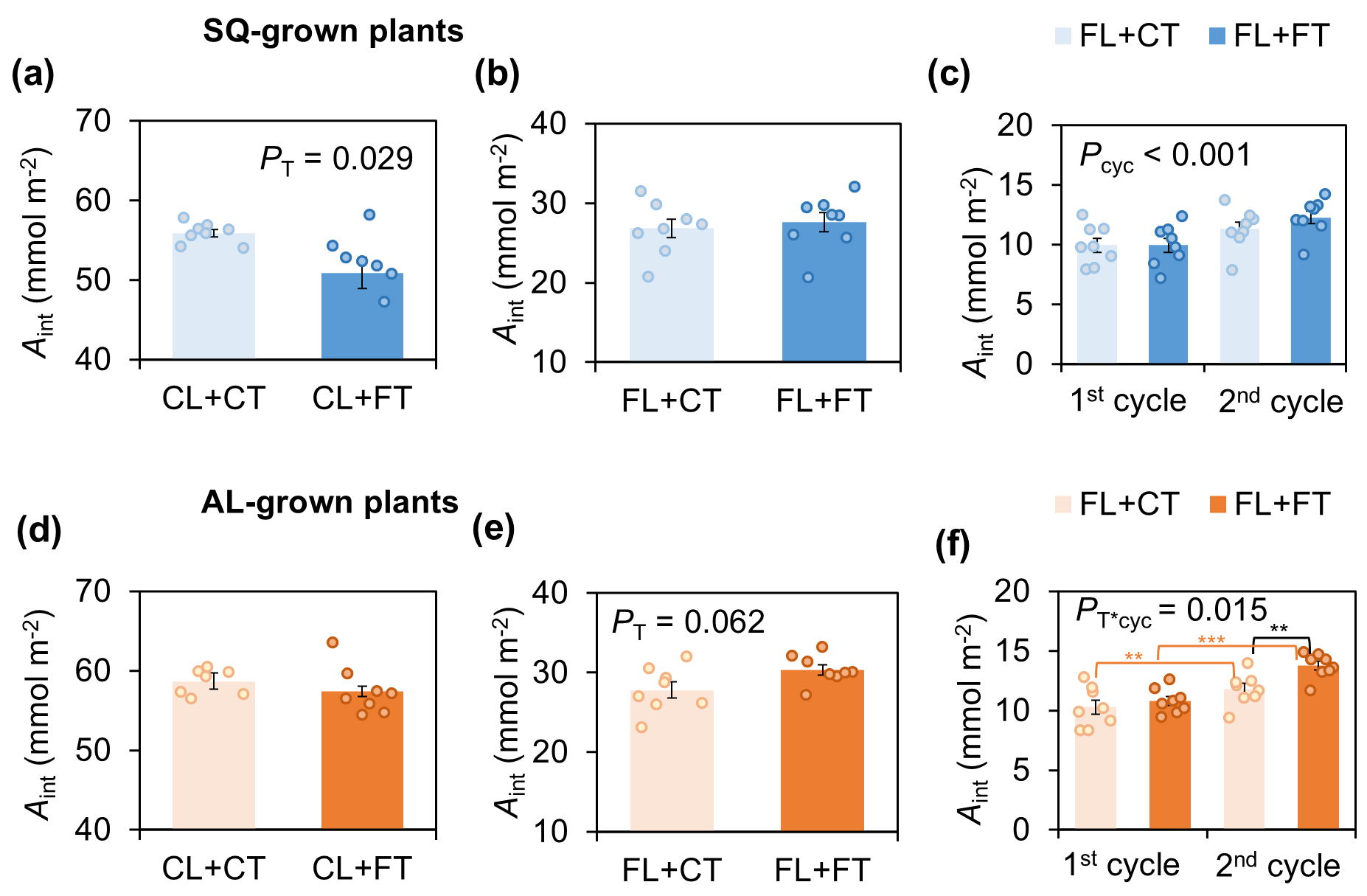


Fig. S9 Effect of T_leaf_ on integrated *A* (*A*_int_) between constant light + constant T_leaf_ (CL+CT) and constant light + fluctuating T_leaf_ (CL+FT) and between fluctuating light + constant temperature (FL+CT) and fluctuating light + fluctuating temperature (FL+FT) for cucumber plants grown under square wave light (SQ; a-c) and alternating light (AL; d-f). Comparison of *A*_int_ over the full measurement time course for all four gas exchange measurement protocols (a, b, d, e) and over the respective cycles of measurement for fluctuating light and/or T_leaf_ (c, f). Data represent means ± SEM (n=8). *P*-value of the main effect of temperature (*P*_T_), measurement cycle (*P*_cyc_), and the interaction effect between temperature and measurement cycle (P_T*cyc_) are shown. Asterisks indicate the significant pairwise comparisons within the levels of the interacting factors: ** P < 0.01, and *** P < 0.001.


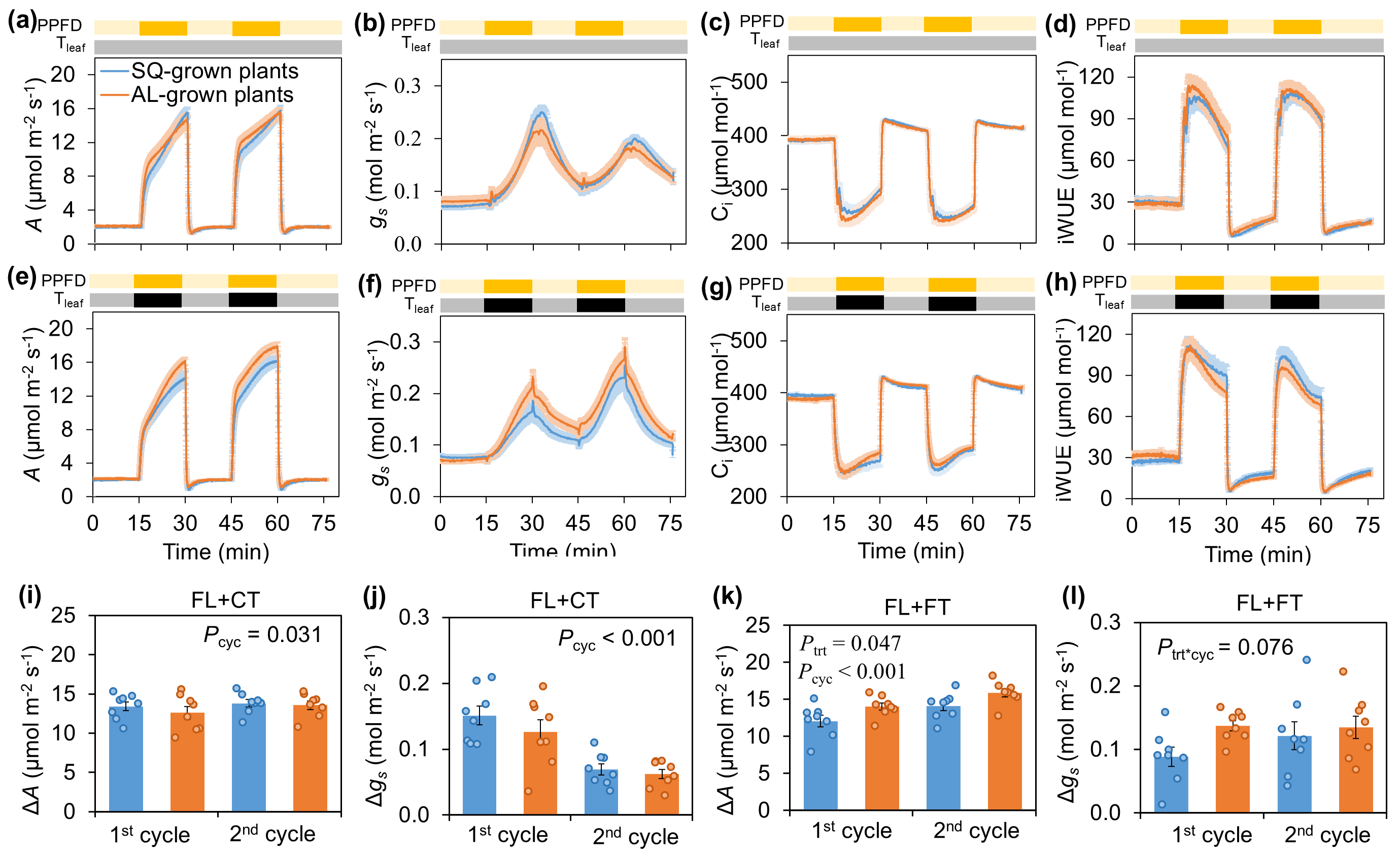


Fig. S10 Gas exchange responses of cucumber leaves grown under square wave (SQ) and alternating light (AL) measured under two protocols: fluctuating light + constant temperature (FL+CT) and fluctuating light + fluctuating temperature (FL+FT). Temporal response of *A*, g_s_, C_i_, and iWUE measured under FL+CT (a-d) and FL+FT (e-h). Changes in *A* (Δ*A*) and g_s_ (Δg_s_) under FL+CT (i, j) and FL+FT (k, l). Yellow and gray bars above panels represent PPFD and T_leaf_, respectively. Bars with uniform color represent constant T_leaf_, bars alternating between light and dark shades indicate fluctuations in PPFD and/or T_leaf_. Data represent means ± SEM (n=8). *P*-value of the main effect of growth treatment (*P*_trt_), measurement cycle (*P*_cyc_), and the interaction effect between growth treatment and measurement cycle (*P*_trt*cyc_) are shown.


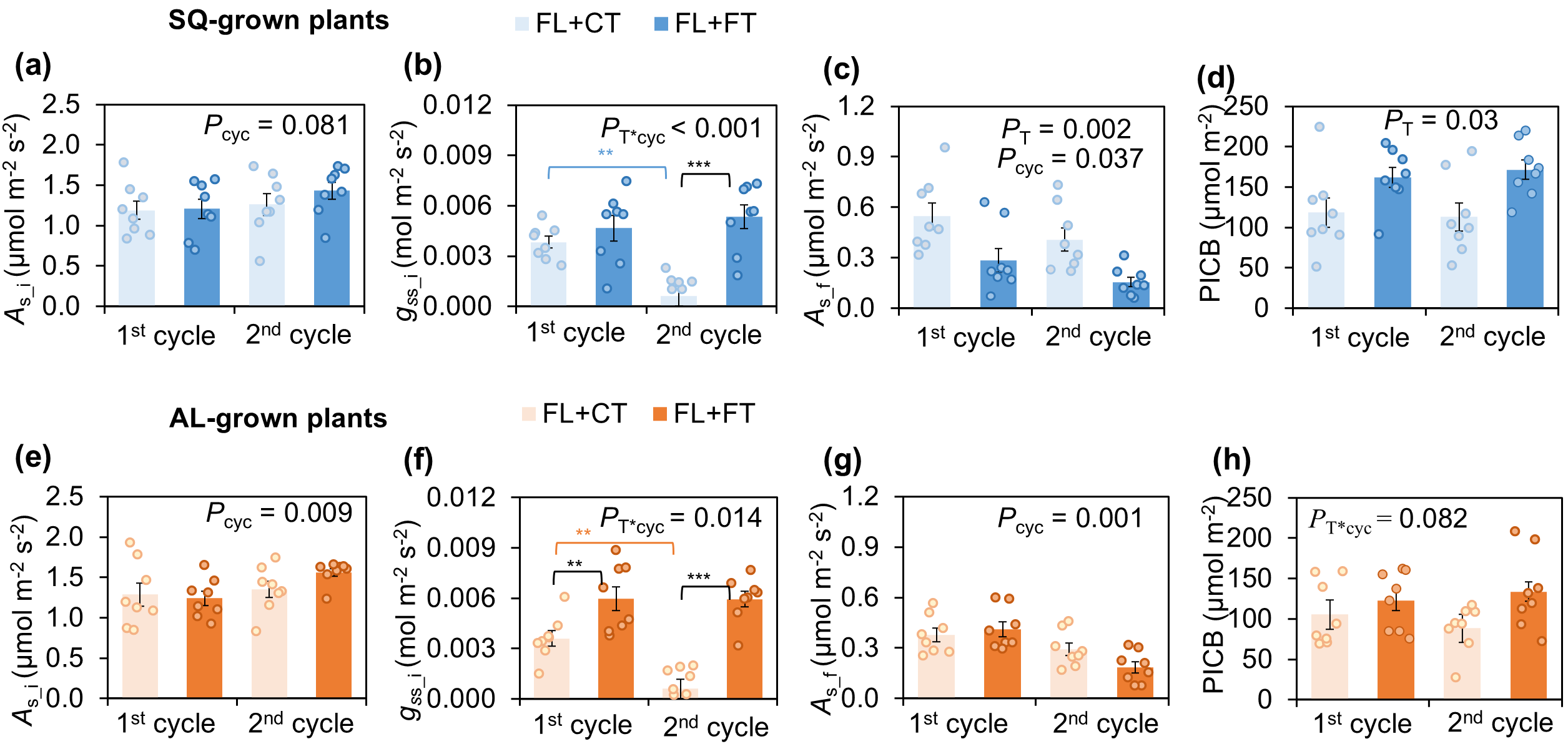


Fig. S11 Effects of T_leaf_ on rate of *A* and g_s_ increase as estimated by slope and post-illumination CO_2_ burst (PICB) between the measurement protocols: fluctuating light + constant temperature (FL+CT) and fluctuating light + fluctuating temperature (FL+FT) for cucumber plants grown under square wave light (SQ; a-d) and alternating light (AL, e-h). Slope of the initial 5 min after PPFD and/or T_leaf_ increase for *A* (*A*_s_i_) and g_s_ (*g_s_*_s_i_) and slope of the final 5 min before PPFD and/or T_leaf_ decrease (*A*_s_f_) for SQ-grown plans (a-c) and AL-grown plants (e-g). PICB for SQ-grown plants (d) and AL-grown plants (h). Data represent means ± SEM (n=8). *P*-value of the main effect temperature (*P*_T_), measurement cycle (*P*_cyc_), and the interaction effect between temperature and measurement cycle (P_T*cyc_) are shown. Asterisks indicate the significant pairwise comparisons within the levels of the interacting factors: ** P < 0.01, and *** P < 0.001.
